# Design and Visualization of a Hierarchical Metamaterial with Tunable Stiffness

**DOI:** 10.34133/research.0874

**Published:** 2025-10-16

**Authors:** Kaili Xi, Xiaoyi Jiang, Dechen Zhao, Guimin Chen, Jiayao Ma, Yan Chen

**Affiliations:** ^1^ School of Mechanical Engineering, Tianjin University, Tianjin 300350, China.; ^2^ State Key Laboratory for Manufacturing Systems Engineering and Shaanxi Key Lab of Intelligent Robots, Xi’an Jiaotong University, Xi’an, China.; ^3^ Key Laboratory of Mechanism Theory and Equipment Design of Ministry of Education, Tianjin University, Tianjin 300350, China.

## Abstract

Metamaterials with stiffness tunability have demonstrated great potential in mechanical systems that operate in variable environments. However, most existing stiffness-tunable metamaterials exhibit high-dimensional and nonlinear structure–property relationships, which hinder precise on-demand tuning and real-time stiffness visualization. Here, we present a reconfigurable hierarchical metamaterial that integrates stiffness tunability, linear structure–property relationship, and real-time self-sensing stiffness visualization into a unified platform. Kinematic analysis shows that the hierarchical metamaterial can be reconfigured through kinematic bifurcations into various single- and multi-level configurations with different numbers of active hinges, and maintain a single degree of freedom in each deformation path. Subsequently, a linear relationship between the number of active hinges and the stiffness of the metamaterial is established through theoretical modelling and verified by experiments, allowing a wide range of tunable stiffness. Furthermore, mechanical logic circuits are embedded into the metamaterial to map transitions between units at different levels to electrical outputs, achieving real-time stiffness visualization via light-emitting diode (LED) states without external sensors. This proposed metamaterial, and more generally, the structure–property–information integration design framework, will greatly advance the development of intelligent adaptive systems.

## Introduction

Stiffness, a fundamental property of materials, plays a critical role in various applications, ranging from vibration isolators [1,2] to robotics [3,4] to wearable devices [5,6]. Metamaterials, which are artificial materials with deliberately designed units [7–11], provide a suitable platform for achieving various stiffness properties, including ultra-high stiffness [12,13], negative stiffness [14–16], and programmable stiffness [17–19]. Among them, metamaterials with tunable stiffness [20,21] can perform multiple operational tasks and adapt to different environments, offering substantial potential for applications in smart structures, robots, and other systems that need to operate in variable environments [22–24].

To achieve tunable stiffness in metamaterials post-fabrication, a common approach is to change their base material properties by external field stimuli, such as thermal [25,26] or magnetic [27,28] fields, allowing a wide range of continuously tunable stiffness. For example, by using a thermo-responsive shape memory polymer that exhibits a substantial change in elastic modulus with temperature variation, mechanical metamaterials with stiffness variations of more than 100 times can be created [29]. In addition, a magnetically shape-shifting kirigami dome metamaterial with high deformability can respond to changing magnetic fields, enabling precise and tunable stiffness control [30]. However, metamaterials designed using this approach generally require a continuous energy supply from an external system to maintain the target stiffness.

Beyond external stimuli, tunable stiffness can be achieved by altering the structural configurations of the metamaterial units. Bistable or multi-stable units, which exhibit varying stiffness in different stable states [31–33], have been widely used in the design of tunable stiffness metamaterials. For example, by selectively controlling beam-based bistable units in a metamaterial to switch between their 2 stable states, a stiffness change of more than 30 times can be achieved [34]. Multi-stable units, such as those inspired by cat-tongue barbs, can also be assembled into arrays to provide a wide range of tunable stiffness values [35]. Reconfiguring metamaterial units to change their deformation paths provides another approach for tunable stiffness [36,37]. Origami or kirigami units, with their rich reconfiguration paths, enable metamaterials to switch between various configurations to modulate the stiffness [38–40]. Gear-based units with anisotropic stiffness can also be used to design tunable stiffness, where the orientation of the units can be adjusted through gear rotation and transfer [41]. However, despite considerable progress in achieving tunable stiffness, a critical challenge remains: the high-dimensional and nonlinear relationship between the deformation of tunable structures and their overall properties, which hinders precise on-demand tuning [34,42] as it often requires complex inverse parameter solutions.

In addition to tunable stiffness, real-time stiffness visualization, which translates the stiffness information into digital outputs, allows users to know the exact stiffness after tuning without the need for external sensors, and thus holds great application potential in human–robot interaction and adaptive robotic systems [43,44]. The key challenge to achieve real-time stiffness visualization is how to establish a simple and robust mapping between structure, properties, and information. Recent studies of the structure–information relationship have enabled metamaterials to process information [45–47] and achieve information display [48,49] using their inherent characteristics, but the integration of these 3 elements remains unexplored due to the nonlinear relationship between structural states and properties of the metamaterials with tunable stiffness.

To address these challenges, inspired by the hierarchical metamaterials [50,51], we propose a metamaterial that can change stiffness over a wide range through reconfiguring into configurations at different hierarchical levels. We establish a linear relationship between the stiffness of the hierarchical metamaterial and the number of active hinges existing in the configuration, thereby linearizing the structure–property relationship. Furthermore, we embed mechanical logic circuits [48,52] into the metamaterial to translate configuration levels into LED-visualized stiffness values, thus realizing the integration of structure, property, and information. Our approach enables a wide range of tunable stiffness and real-time stiffness visualization without the need for external sensors, paving the way for intelligent systems with self-sensing and adaptive capabilities.

## Results

### Geometry, reconfigurability, and hierarchy of the metamaterials

**Fig. 1.**
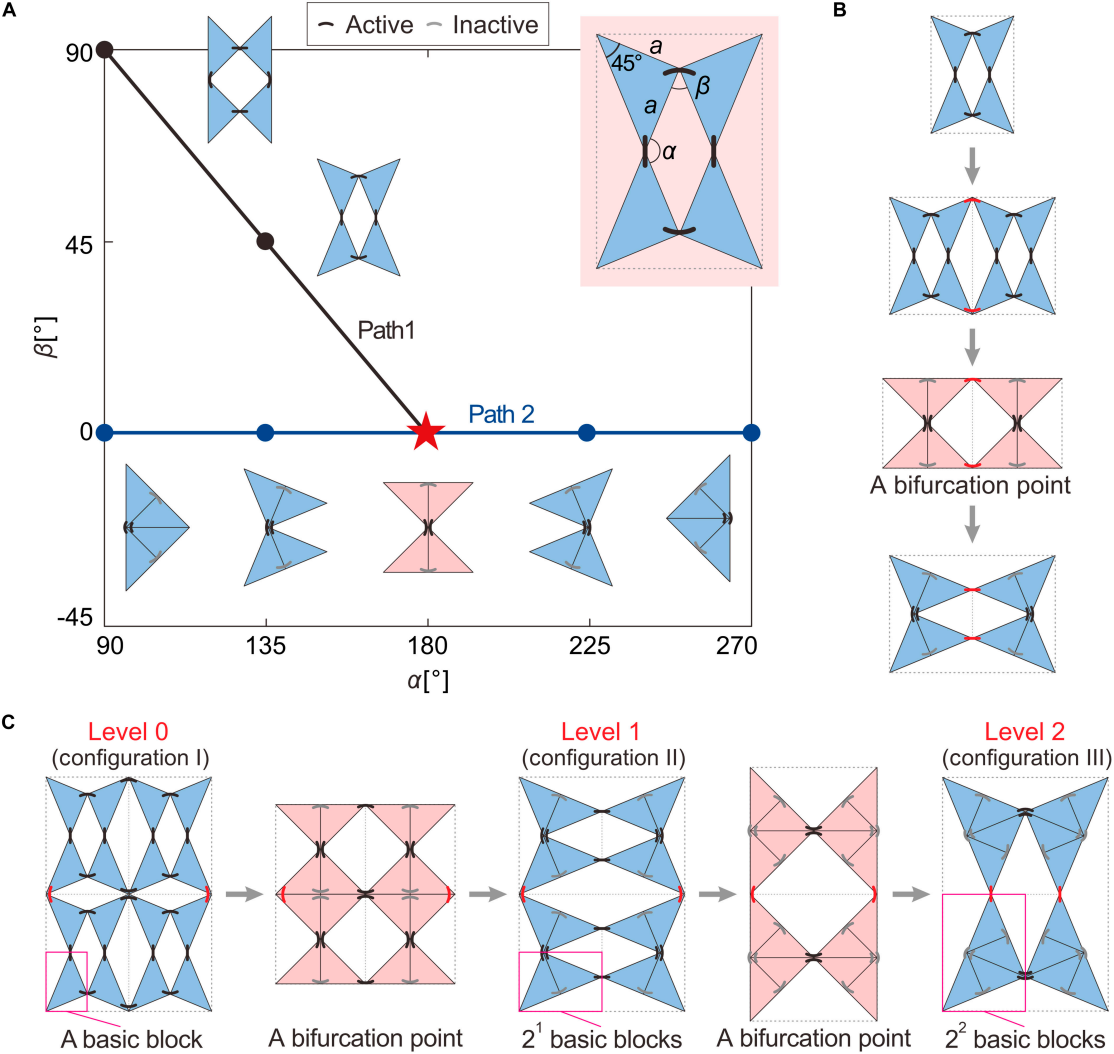
Design of reconfigurable metamaterials and their hierarchical levels. (A) Unit cell and its 2 motion paths, with typical configurations on each path. The unit cell consists of 4 isosceles right-angled triangular blocks, each with a waist length *a* = 15 mm and thickness *t* = 20 mm, connected by 4 hinges (black arcs). The kinematics of the unit cell is determined by 2 kinematic variables, *α* and *β*. It has a bifurcation point at *α* = 180° and *β* = 0°, and the corresponding configuration is highlighted in pink. (B) Construction of a 1 × 2 metamaterial and its reconfiguration process. Black arcs represent hinges within the unit, while red arcs represent hinges connecting units in the tessellation. (C) Reconfiguration of the 2 × 2 metamaterial between configurations I, II, and III, which are defined as a single-level configuration with 4 level 0 units, 2 level 1 units, and 1 level 2 unit, respectively. Each triangular block in the level *i* unit consists of 2*^i^* basic blocks.

The metamaterial unit, as shown in Fig. 1A, is composed of 4 isosceles right-angled triangular blocks with waist length *a* and thickness *t*, connected by 4 hinges (black arcs), which form a planar 4-bar linkage. A kinematic analysis of the unit indicates that it is a single degree of freedom (DOF) and bifurcates at *α* = 180° and *β* = 0°, where *α* and *β* are dihedral angles, thus leading to 2 distinct motion paths. On path 1 (the black line), the unit behaves as a planar 4-bar linkage with all 4 hinges active during motion following the relationship *β* = 180° − *α*. At the bifurcation point marked by a star, the upper and lower pairs of adjacent triangular blocks contact each other to form new, larger blocks that are geometrically similar to the initial ones. The unit then transitions from a 4-bar linkage to a 2-bar linkage, also with a single DOF, and moves along path 2 (the blue line) where *α* varies from 90° to 270°, while *β* remains at 0°. During this motion, the 2 hinges enclosed in the larger blocks do not rotate and are thus considered inactive (gray arcs), leaving only 2 active hinges.

This kinematic bifurcation behavior of the unit, when tessellated in a plane, allows the metamaterial to reconfigure into a variety of configurations and form hierarchies. To illustrate this, first consider the simplest case when 2 units are connected by 2 additional hinges (red arcs) to form a 1 × 2 metamaterial, as shown in Fig. 1B. Initially, the metamaterial can be considered as an assembly of two 4-bar linkages and still moves with a single DOF. When it reaches the bifurcation point where each unit becomes a 2-bar linkage, it transitions into a planar 4-bar linkage like the unit, but now each large triangular block consists of 2 basic ones. After reconfiguration, the number of active hinges is reduced from 10 to 4. Further extending the metamaterial to 2 × 2 by connecting two 1 × 2 metamaterials with 2 hinges (red arcs) as shown in Fig. 1C, it can still preserve the single DOF motion and kinematic bifurcation (detailed derivation in Note S1). Therefore, the 2 × 2 metamaterial first reconfigures into an assembly of two 4-bar linkages in which each triangular block comprises 2^1^ basic ones, and then into a single 4-bar linkage where each triangular block comprises 2^2^ basic ones. Correspondingly, we can define the hierarchy of the metamaterial based on the size of triangular blocks in the 4-bar linkages. Still taking the 2 × 2 metamaterial as an instance, at configuration I, only basic triangular blocks exist, and thus, the metamaterial has a single-level configuration with 4 level 0 units. At configuration II, each triangular block is formed by 2^1^ basic ones, and the metamaterial still has a single-level configuration with 2 level 1 units. Similarly, at configuration III, the metamaterial has a single-level configuration with one level 2 unit, as each block is composed of 2^2^ basic ones. The number of active hinges at the 3 levels is respectively 22, 14, and 6. It is worth noting that the metamaterials can actually generate more motion paths and bifurcation points than we have presented in Fig. 1, which are not discussed here as they are not closely related to the hierarchical levels mentioned above. See more details in Movie S1 and Note S1.

More generally, a $2^{\left\lfloor i/2\right\rfloor }\times 2^{\left\lfloor \left(i+1\right)/2\right\rfloor }$ metamaterial, constructed by 2*^i^* level 0 units, can be continuously reconfigured from level 0 to level *i* (*i* > 0), thus forming *i* + 1 single-level configurations, where ⌊*i*/2⌋ and ⌊(*i* + 1)/2⌋ denote the floor function (representing the largest integer less than or equal to the given value). We have recursively proven that these hierarchical metamaterials maintain a single DOF throughout their motion paths except at bifurcation points (Note S1).

**Fig. 2.**
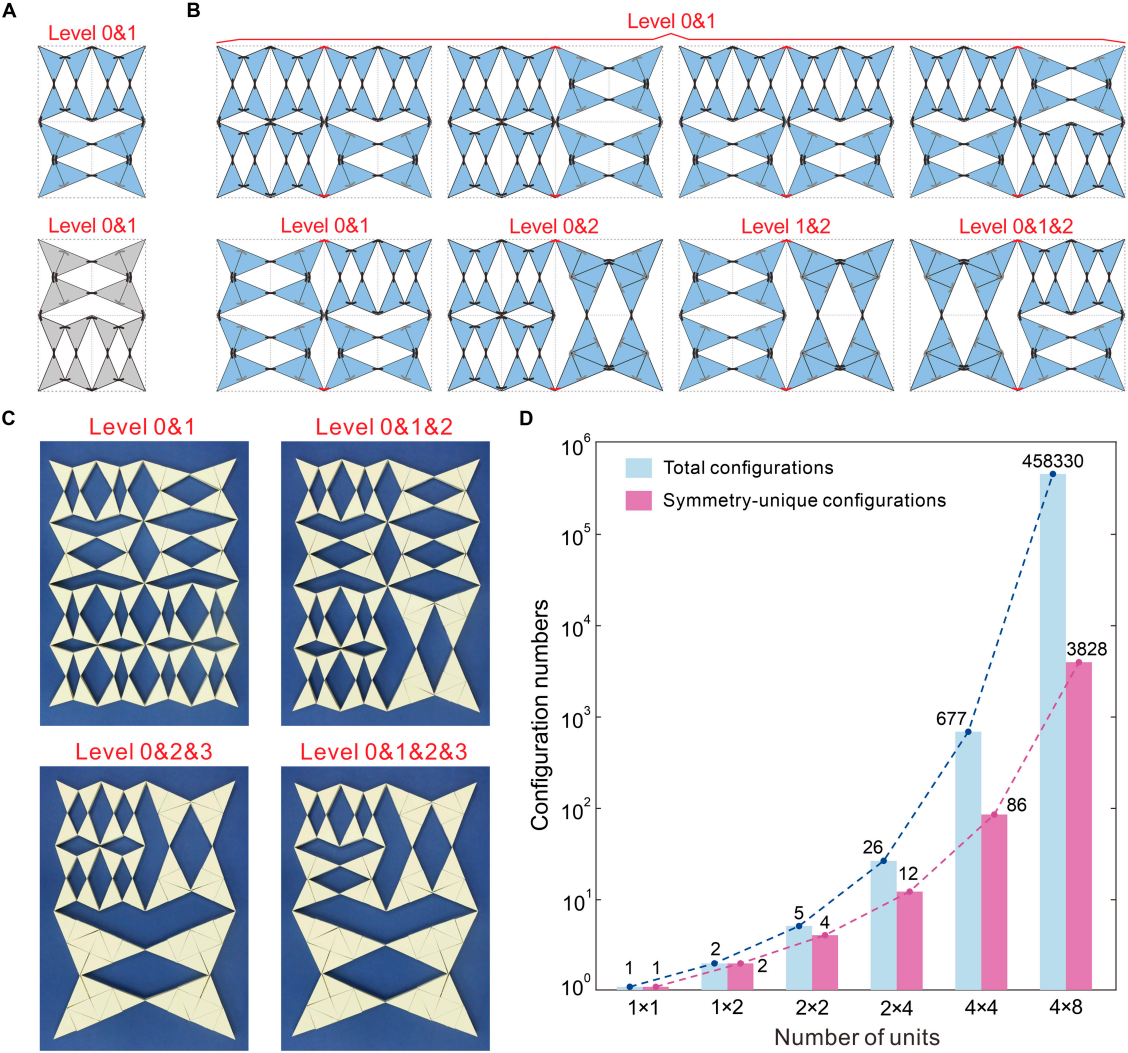
Reconfiguration analysis of the multi-level configurations. (A) Two multi-level configurations of the 2 × 2 metamaterial with 2 level 0 units and 1 level 1 unit. Only the blue configuration is distinct, as the gray one can be obtained by vertically mirroring the blue one. (B) Eight distinct multi-level configurations of the 2 × 4 metamaterial: 5 level 0&1 ones varying numbers of level 0 and level 1 units, 1 level 0&2, 1 level 1&2, and 1 level 0&1&2. (C) Paper model of the 4 × 4 metamaterial in 4 multi-level configurations: levels 0&1, 0&1&2, 0&2&3, and 0&1&2&3. (D) Relationship between the total number of metamaterial configurations and the number of symmetry-unique configurations with the number of units.

Beyond single-level configurations, the metamaterial can also be reconfigured to contain units of different levels, thus forming various multi-level configurations. To explore this, we recursively analyze the number of multi-level configurations and the correlation with the number of units. It is obvious that the 1 × 2 metamaterial in Fig. 1B can only form single-level configurations, and thus, we start from the 2 × 2 metamaterial in Fig. 1C. If the 2 level 0 units at the bottom are reconfigured to one level 1 unit, while the other 2 at the top remain unchanged, a multi-level configuration with 18 active hinges featuring 2 level 0 units and one level 1 unit can be obtained (the blue one in Fig. 2A). Alternatively, reconfiguring the top 2 level 0 units into one level 1 unit while keeping the bottom 2 units unchanged yields another multi-level configuration with the same number of active hinges (the gray configuration in Fig. 2A). Since the gray configuration can be obtained by vertically mirroring the blue configuration, they are symmetry-equivalent [53] and expected to yield identical stiffness. Therefore, in this study, we exclude configurations that are symmetry-equivalent by symmetry operations (second-order rotational symmetry, vertical mirroring, or horizontal mirroring), using a developed screening procedure. See Note S2 and Supplementary Code for details. We retain only one representative configuration from each set of mutually symmetry-equivalent configurations, thereby identifying the symmetry-unique configurations. Thus, the 2 × 2 metamaterial has only one symmetry-unique configuration. For the 2 × 4 metamaterial, its multi-level configurations can be derived from the 2 × 2 metamaterial. Specifically, the 3 single-level and 2 multi-level configurations of the 2 × 2 metamaterial can be combined to form $C_{5}^{1}C_{5}^{1}=5^{2}=25$ configurations for the 2 × 4 metamaterial. Excluding the 3 single-level configurations (at levels 0, 1, and 2, respectively) constructed from the self-combinations of the single-level configurations, there are 5^2^ − 3 = 22 multi-level configurations. Among them, 8 symmetry-unique configurations are shown in Fig. 2B: 5 level 0&1 configurations with varying numbers of level 0 and level 1 units, 1 level 0&2, 1 level 1&2, and 1 level 0&1&2.

Further, for the 4 × 4 metamaterial, the number of multi-level configurations is deduced from the 2 × 4 metamaterial. Through a similar analysis, it is found to have (22 + 4)^2^ − 4 = 672 multi-level configurations, among which 81 are symmetry-unique ones. Figure 2C illustrates the paper prototypes of 4 representative multi-level configurations: 0&1, 0&1&2, 0&2&3, and 0&1&2&3.

To generalize, we denote the total number of multi-level configurations for a $2^{\left\lfloor i/2\right\rfloor }\times 2^{\left\lfloor \left(i+1\right)/2\right\rfloor }$ metamaterial with the highest level *i* as *P_^i^_*, given by:

$$\begin{array}{l} P_{0}=P_{1}=0,\\ P_{2}=2,\\ \;P_{i}={\left(P_{i-1}+i\right)}^{2}-i,\left(i\geq 2\right) \end{array}.$$ (1)

Adding the number of single-level configurations, the total number of configurations for metamaterials can be expressed as *P_i_* + *i* + 1, where the number of symmetry-unique configurations can be determined by an automated screening procedure (Note S2). Figure 2D illustrates the exponential increase in the number of total and symmetry-unique configurations from 1 × 1 to 4 × 8 metamaterials. For instance, a 4 × 8 metamaterial would have 458,330 configurations, including 3,828 symmetry-unique configurations, fully demonstrating the reconfigurability of these hierarchical metamaterials.

Finally, all the configurations, including both the single-level and multi-level ones, can be mutually transferred through kinematic bifurcation, and the resultant metamaterial maintains a single DOF motion except for the bifurcation points (detailed derivation in Note S1). As an instance, the reconfiguration process of a 4 × 4 metamaterial among 10 configurations (5 single-level and 5 multi-level) is demonstrated in Movie S2.

### Tunable stiffness in hierarchical metamaterials

**Fig. 3.**
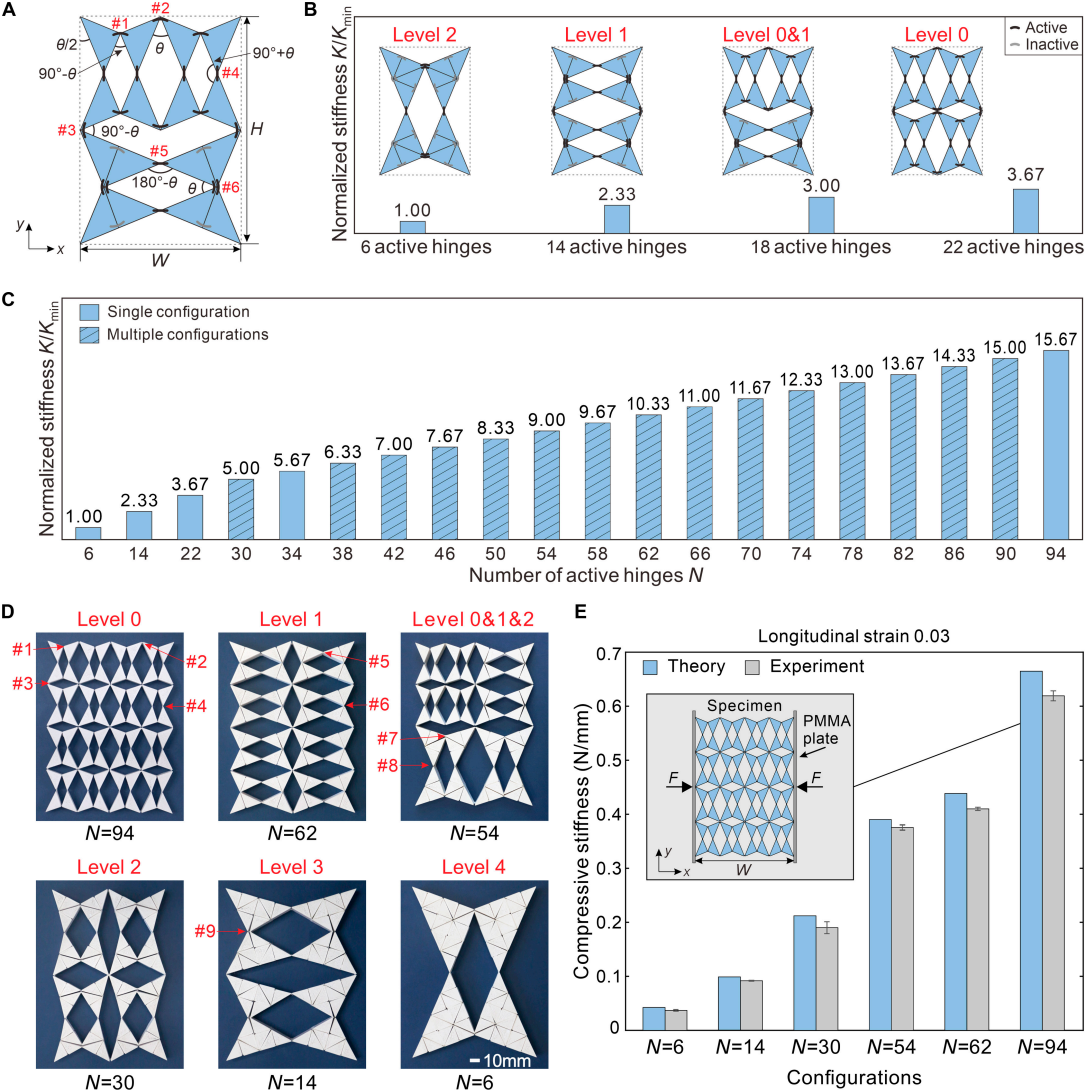
Tunable stiffness in hierarchical metamaterials. (A) Angles of 6 types of hinges in the multi-level configuration of the 2 × 2 metamaterial, with 2 level 0 units and 1 level 1 unit. (B) Normalized stiffness of the 2 × 2 and (C) 4 × 4 metamaterials at different configurations, with distinct numbers of active hinges. The number of active hinges indicated by the shaded column has multiple configurations. (D) Specimens of the 4 × 4 metamaterial in 6 configurations (levels 0, 1, 0&1&2, 2, 3, and 4) with 94, 62, 54, 30, 14, and 6 active hinges, respectively. The 9 types of hinges (#1 to #9) are marked in red. (E) Comparison of theoretical and experimental compressive stiffness of 6 configurations in (D) under 3% strain.

The hierarchical reconfiguration, which leads to the variation of active hinges and single DOF motion behavior of the metamaterial, enables tunable stiffness over a wide range. Without losing generality, first consider the 2 × 2 metamaterial at the multi-level configuration as shown in Fig. 3A to illustrate this. Defining the angle between the vertical side of the metamaterial outer contour and the triangular block as *θ*/2, the active hinges of the metamaterial can be categorized into 6 groups (#1 to #6) with dihedral angles of 90° − *θ*, *θ*, 90° − *θ*, 90° + *θ*, 180° − *θ*, and *θ*, respectively. Among these, hinges #1 to #4 are the 4 basic hinges in the initial printed configuration of the metamaterial (Fig. S7). Hinge #5 is reconfigured from hinge #2, and hinge #6 is formed by merging 2 hinges (#4). The other 3 hinge types (#7 to #9) discussed in this paper can also be derived from the 4 basic hinges through reconfiguration or merging processes (see Notes S4 and S5 and Fig. S8 for details). When compressed along the *x* axis, governed by the single DOF motion kinematics, all the active hinges rotate by the same magnitude *∆θ*. Since the triangular blocks are much stiffer than the hinges, the strain energy of the metamaterial, *U*, can be calculated as the summation of the strain energy of the hinges. Subsequently, the stiffness can be obtained by taking the second derivative of the strain energy with respect to the width $W=2a\cos\left(\pi /4-\theta /2\right)$ of the metamaterial as follows

$$K=\frac{d^{2}U}{{\mathrm{dW}}^{2}}=\frac{\left(2+\text{cot}\left(\pi /4-\theta /2\right)\Delta \theta \right)}{8a^{2}{\text{sin}}^{2}\left(\pi /4-\theta /2\right)}\mathrm{Nk},$$ (2)

in which *N* is the number of active hinges and *k* is the rotational stiffness of each hinge. This rotational stiffness was measured after 14 rounds of thermal reconfiguration to ensure consistent performance, since the stiffness of the hinges stabilizes after this initial period (see Fig. S9 and Note S5 for details). For a given metamaterial, if *θ* is maintained when it is reconfigured to different levels so as to keep its outer contour unchanged, Eq. (2) is always applicable. Therefore, the stiffness in a particular configuration is only linearly correlated to the number of active hinges, provided that the hinge rotational stiffness is not affected by reconfiguration. Figure 3B presents all 4 configurations of the 2 × 2 metamaterial at different levels, which have 6, 14, 18, and 22 active hinges, respectively. Correspondingly, the stiffness can be tuned by a ratio of 3.67, the ratio of the maximum stiffness to the minimum stiffness.

More generally, the above conclusion can be extended to a $2^{\left\lfloor i/2\right\rfloor }\times 2^{\left\lfloor \left(i+1\right)/2\right\rfloor }$ metamaterial, which is constructed from 2*^i^* level 0 units and has a total of *N*_T_ = 3 × 2^*i* + 1^ − 2 hinges (Note S1). This metamaterial can be reconfigured into configurations composed of various units at different levels, where the number of level *j* units is denoted as *u*_*j*​_ (*j* varies from 0 to *i* and *u*_*j*​_ ≤ 2^*i* – *j*^; details in Note S3). Given that inactive hinges only exist in level *j* units (*j* ≥ 1), the number of active hinges in this metamaterial is determined by subtracting the inactive hinges contributed by each level *j* unit (*j* ≥ 1). To distinguish from the overall metamaterial, we define the total number of hinges in a level *j* (*j* ≥ 1) unit as *n*_*j*,T_, and the number of inactive hinges in that unit as *n*_*j*,inact_. Specifically, a level *j* unit has a total of *n*_*j*,T_ = 3 × 2^*j* + 1^ − 2 hinges, but only 6 of them are active (Note S1), resulting in *n*_*j*,inact_ = 3 × 2^*j* + 1^ − 8 inactive hinges. Therefore, the number of active hinges of the metamaterial can be calculated as

$$N=3\times 2^{i+1}-2-\sum_{1}^{i}\left(3\times 2^{j+1}-8\right)u_{j},$$ (3)

Using the same approach, the compressive stiffness of the $2^{\left\lfloor i/2\right\rfloor }\times 2^{\left\lfloor \left(i+1\right)/2\right\rfloor }$ metamaterial in the *x* direction can also be derived as follows

$$K=\frac{d^{2}U}{{\mathrm{dW}}^{2}}=2^{-\left(2\left\lfloor \left(i+1\right)/2\right\rfloor +1\right)}\frac{\left(2+\text{cot}\left(\pi /4-\theta /2\right)\Delta \theta \right)}{a^{2}{\text{sin}}^{2}\left(\pi /4-\theta /2\right)}\mathrm{Nk}.$$ (4)

The stiffness tunability ratio of the metamaterial can be calculated as

$$\frac{K_{\max}}{K_{\min}}=\frac{N_{\max}}{N_{\min}}=2^{i}-1/3,$$ (5)

where *N*_max_ = *N*_T_ = 3 × 2^*i* + 1^ − 2 is the total number of hinges in the metamaterial (all units at level 0) and *N*_min_ = 6 corresponds to the metamaterial at the single-level configuration with one level *i* unit. The stiffness tunability ratio is proportional to 2*^i^*, the number of level 0 units.

Moreover, the resolution of the tunable stiffness, *R*, which represents the difference between 2 neighboring stiffness values, is determined by the increase in inactive hinges when 2 level *j* units are reconfigured into one level *j* + 1 unit.

R=nj+1,inact−2nj,inact=Rmin=4,j=0Rnrm=8,j≥1. (6)

Finally, the compressive stiffness of the metamaterial in the *y* direction can also be obtained following the same procedure, which also shows a linear correlation with the number of active hinges (Note S3).

When the size of the metamaterial reaches 4 × 4 (*i* = 4), it includes 86 configurations with 20 different active hinge numbers of 6, 14, 22, 30, 34, 38, 42, 46, 50, 54, 58, 62, 66, 70, 74, 78, 82, 86, 90, and 94, leading to a wider tunability ratio of 15.67 (Fig. 3C). Further extending the metamaterial to 16 × 16 (*i* = 8) will result in a stiffness tunability ratio of 255.67 [Eq. (5)].

**Fig. 4.**
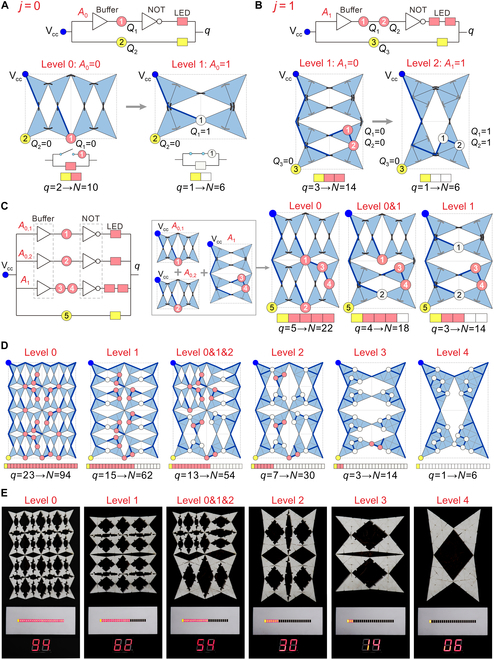
Visualization of the number of active hinges in hierarchical metamaterials with tunable stiffness. (A) Design schematic for the metamaterial reconfiguring from 2 level 0 units into 1 level 1 unit, including a buffer gate and a NOT gate. These 2 configurations are abstracted as the binary mechanical inputs *A*_0_, where *A*_0_ = 0 for level 0 and *A*_0_ = 1 for level 1. The power input terminal is marked by a blue dot, the outputs *Q*_1_ and *Q*_2_ are marked by nodes ① and ②, and LEDs are marked by rectangles. Here, *q* denotes the number of lit LEDs, with each pink LED representing 4 active hinges and the yellow LED representing 6 active hinges. Circuits attached to the surface of the metamaterial (marked by navy lines) implement the buffer gate. At level 0 (*A*_0_ = 0), output node ① is not connected, and *Q*_1_ = 0; at level 1 (*A*_0_ = 1), the output node ① is connected, and *Q*_1_ = 1. The output *Q*_1_ is used as an input for the NOT gate: When *Q*_1_ = 0, the pink LED is lit; when *Q*_1_ = 1, the pink LED is shorted and turned off (white). (B) Design schematic for the metamaterial reconfiguring from 2 level 1 units to 1 level 2 unit. (C) Logic diagram and schematic to visualize the number of active hinges for 2 × 2 metamaterials, from the single-level configuration to the multi-level configuration. Outputs ① to ⑤ control a row of LEDs to show the number of active hinges, which correlates linearly with the number of active hinges *N*. (D) Schematic to visualize the number of active hinges for 4 × 4 metamaterials, for 6 typical configurations with 94, 62, 54, 30, 14, and 6 active hinges, respectively. (E) Experimental results of the 4 × 4 metamaterial in 6 configurations of (D). The LED row indicates the number of lit LEDs *q*, while the 2-digit 7-segment display shows the number of active hinges *N*.

The 4 × 4 metamaterial was designed, fabricated, and tested to validate the theoretical model. Three-dimensional (3D) printing was adopted to manufacture the specimens with thermoplastic polyurethane (TPU) (Note S4). Quasi-static uniaxial compression tests were conducted on 6 typical configurations—levels 0, 1, 0&1&2, 2, 3, and 4—with 94, 62, 54, 30, 14, and 6 active hinges, respectively (Fig. 4D). The experimental setup is shown in Fig. 3E (see details in Movie S3 and Note S5). The experimental and theoretical stiffness values are compared in Fig. 3E, demonstrating a clear difference in stiffness between these configurations and a good agreement between the theoretical and experimental results. Furthermore, the stiffness of other 4 multi-level configurations (level 1&2, 2 different level 0&1&2, and level 0&3), each with 54 active hinges (Fig. S10C), was experimentally measured and found to be consistent with theoretical predictions (Fig. S10D and E). These results confirm that the theoretical model accurately predicts the stiffness of metamaterials.

### Real-time stiffness visualization

To visualize the stiffness of the hierarchical metamaterial in real time, we integrate reconfigurable circuits in the metamaterial to map the transitions among units at different levels to the multichannel electrical outputs. This establishes a linear relationship between structure, properties, and information, enabling the metamaterial to display the number of active hinges and stiffness in various configurations without the need for external sensors. To illustrate this, we first consider the reconfiguration between single-level configurations from 2 level *j* units to 1 level *j* + 1 unit, during which the reduction in the number of active hinges *N* can be obtained from Eq. (6). These 2 configurations can be abstracted as the binary mechanical inputs *A_j_*, where *A_j_* = 0 for level *j* and *A_j_* = 1 for level *j* + 1, to obtain the corresponding electrical outputs. When *j* = 0 (Fig. 4A), a logic operator is designed for this reconfiguration, including a buffer gate and a NOT gate, where the power input terminal V_cc_ and the electrical outputs *Q*_1_ and *Q*_2_ are represented by the blue dot and nodes ① and ②, respectively. Each pink LED (marked by rectangles) indicates that 4 hinges are active when it is lit, and the yellow LED is always lit as the minimum number of active hinges for a metamaterial is *N*_min_ = 6. By defining the number of lit LEDs as *q*, the number of active hinges *N* can be indicated by *q*. The circuits to implement the buffer gate are marked by navy lines on the surface of the metamaterial. At level 0 (*A*_0_ = 0), the basic triangular blocks in the metamaterial are discrete. Therefore, the output node ① is not connected, and *Q*_1_ = 0. When the 2 level 0 units are reconfigured into 1 level 1 unit (*A*_0_ = 1), these basic triangular blocks are merged into larger blocks to connect the circuits across different blocks. This connection activates the output node ①, and thus, *Q*_1_ = 1. The output *Q*_1_ is then used as the input to the NOT gate. When *Q*_1_ = 0, both the pink and yellow LEDs are lit, and *q* = 2, which indicates 10 active hinges. When *Q*_1_ = 1, the pink LED is shorted and turned off (white), and only the yellow LED is still on, with *q* = 1 and 6 active hinges. When *j* = 1 (Fig. 4B), reconfiguring 2 level 1 units into 1 level 2 unit reduces the number of active hinges by 8, thus requiring 2 pink LEDs. Figure 4B shows a similar logic operator consisting of a buffer gate and a NOT gate, yielding 3 outputs *Q*_1_ to *Q*_3_ (represented by nodes ① to ③), where *Q*_1_ and *Q*_2_ are identical. At level 2 (*A*_1_ = 1), 2 output nodes ① and ② are activated (*Q*_1_ = *Q*_2_ = 1), which shorts the 2 pink LEDs. This changes the number of lit LEDs from 3 to 1, indicating that the number of active hinges *N* in this metamaterial varies from 14 to 6. Note that these are just 2 examples of circuit designs among many possible solutions (see Fig. S11 for details on other feasible circuit arrangements). Further extending to the reconfiguration between the single-level configurations from 2 level *j* units to 1 level *j* + 1 unit (*j* > 1), the logic operators for *j* = 1 with outputs *Q*_1_ to *Q*_3_ can still be applied.

In addition to single-level configurations, this method is also applicable to multi-level configurations of metamaterials. For ease of description, if the metamaterial has multiple inputs *A_j_* for the same level *j*, they are notated sequentially as *A*_*j*,*s*_. Taking the 2 × 2 metamaterial as an example, its logic diagram (Fig. 4C) contains 3 pairs of buffer and NOT gates. The first 2 pairs serve as logic operators for *j* = 0 (Fig. 4A) with inputs *A*_0,1_ and *A*_0,2_, respectively, while the third pair serves as the logic operator for *j* = 1 (Fig. 4B) with input *A*_1_. The 3 pairs use a common yellow LED. This design thus results in 5 outputs *Q*_1_ to *Q*_5_ (represented by nodes ① to ⑤), where outputs *Q*_3_ and *Q*_4_ are identical. According to the logic diagram, the global circuits of this metamaterial can be constructed, where outputs *Q*_1_ to *Q*_4_ (nodes ① to ④) control the 4 pink LEDs, and output *Q*_5_ (node ⑤) controls the yellow LED. The number of active hinges *N* can be encoded through a linear sparse mapping with the number of lit LEDs *q*, as follows:

$$N=N_{\min}+4\left(q-1\right),$$ (7)

where the number of lit LEDs *q* can be calculated by $q=\bar{A_{0,1}}+\bar{A_{0,2}}+2\bar{A_{1,2}}+1$. As also seen in Fig. 4C, the number of active hinges at different configurations, from a single-level configuration at level 0 (*q* = 5, 22 active hinges) to a multi-level one at level 0&1 (*q* = 4, 18 active hinges) and then to a single-level configuration at level 1 (*q* = 3, 14 active hinges), can be shown by the states of LEDs.

More generally, this stiffness visualization method can be extended to a $2^{\left\lfloor i/2\right\rfloor }\times 2^{\left\lfloor \left(i+1\right)/2\right\rfloor }$ metamaterial (*i* ≥ 1). The global circuits for the metamaterial are constructed hierarchically by first combining 2^*i* − 1^ logic operators for *j* = 0 with inputs *A*_0,*s*_ (*s* = 1, 2, …, 2^*i* − 1^) to form the initial circuit, and successively superimposing 2^*i* − *j* − 1^ logic operators for each level *j* with inputs *A*_*j*,*s*​_ (*s* = 1, 2, …, 2^*i*−*j*-1^) until *j* = *i* − 1. The global circuits, denoted as *C*_global_, are superimposed as follows

$$C_{\text{global}}=\sum_{0}^{i-1}\sum_{s=1}^{2^{i-j-1}}\text{NOT}\left(\text{buffer}\left(A_{j,s}\right)\right).$$ (8)

Its number of lit LEDs can be expressed as

$$\begin{array}{c} q=\sum_{s=1}^{2^{i-1}}\text{NOT}\left(\text{buffer}\left(A_{0,s}\right)\right)+2\sum_{1}^{i-1}\sum_{s=1}^{2^{i-j-1}}\text{NOT}\left(\text{buffer}\left(A_{j,s}\right)\right)\\ \;+1=\sum_{s=1}^{2^{i-1}}\bar{A_{0,s}}+2\sum_{1}^{i-1}\sum_{s=1}^{2^{i-j-1}}\bar{A_{j,s}}+1. \end{array}$$ (9)

Then, the number of active hinges can be determined using Eq. (7).

The 4 × 4 metamaterial (*i* = 4) was designed, fabricated, and tested to validate this visualization method. As shown in Fig. 4D, the global circuits for this metamaterial are constructed based on Eq. (8) and the logic diagram in Fig. S12A. It generates a maximum of *q*_max_ = 23 LEDs [all units at level 0, see Eq. (7), where *N* = *N*_max_​ = 94 and *N*_min_​ = 6], including 1 highlighted in yellow and the other 22 in pink. Six typical configurations—levels 0, 1, 0&1&2, 2, 3, and 4—with 94, 62, 54, 30, 14, and 6 active hinges, respectively, demonstrate the theoretical results across all hierarchical levels from 0 to 4 (Fig. 4D). The specimen (Fig. S12C to F) integrates 3D-printed TPU structures with conductive elastomer circuits (cast in grooves on the bottom surface) and is reconfigured using snap-fit structures (Note S6). The 7-segment display is used to show the number of active hinges *N*, which is calculated from the number of lit LEDs *q* [see Eq. (7)], with specific implementation details given in Note S6. The experimental results (Fig. 4E and Movie S4) show that the number of active hinges as the 4 × 4 metamaterial reconfigures among 10 configurations (5 single-level and 5 multi-level) can be accurately visualized by the lit LEDs and the 7-segment display, which demonstrate the reliability of the proposed design for real-time stiffness visualization in hierarchical metamaterials without external sensors.

## Conclusion

In summary, we have presented a reconfigurable hierarchical metamaterial that achieves tunable stiffness over a wide range and real-time stiffness visualization without external sensors. We have shown that this metamaterial can be reconfigured into multiple motion paths through kinematic bifurcation, and maintains a single DOF at each path. By exploiting the reconfigurability of the metamaterial, we have established the hierarchical levels at various configurations and obtained the relationship between a configuration and the corresponding number of active hinges that rotate during motion. We have demonstrated through theoretical modelling and experiments that the stiffness of the metamaterial is linearly related to the number of active hinges, thereby achieving a linearized structure–property relationship. Furthermore, we have developed a real-time stiffness visualization method for this hierarchical metamaterial, which utilizes mechanical logic operators to process configuration information and maps it to electrical outputs that indicate stiffness via LED states.

The proposed metamaterial offers 2 major advantages for adaptive systems: (a) Its linear stiffness–hinge relation streamlines modeling and control design, and (ii) its embedded mechanical logic circuits enable real-time stiffness visualization, affording closed-loop stiffness control. These advantages endow the metamaterial with a wide range of potential applications. In robotic joints, for example, the metamaterial can be tuned on demand—to low stiffness for delicate grasping tasks and to high stiffness for heavy-load manipulation—while LED states provide real-time visualization of joint stiffness. In morphing wings, the same metamaterial can cycle among high stiffness for load-bearing cruise, moderate stiffness for fatigue durability, and low stiffness for impact energy dissipation, replacing multi-material assemblies and reducing overall weight [21]. Similarly, medical assistive equipment can use this metamaterial to increase stiffness progressively from low to high, matching the impedance of patients in need of rehabilitation [22]. Overall, this hierarchical metamaterial, which integrates structure, property, and information into a single system, is expected to promote the development of intelligent adaptive systems.

In addition, while our work has been thoroughly validated under quasi-static conditions, dynamic loads are also important in certain applications, such as in robotic joints. In the future, we plan to investigate how the metamaterial responds to dynamic loads, including impact and vibration, to better understand its performance in real-world conditions.

## Materials and Methods

### Manufacture of physical specimens

For the 4 × 4 metamaterial in Fig. S7A, all 94 hinges are active, with triangular blocks of side length *a* = 18 mm and thickness *t* = 20 mm, and the initial configuration at angle *θ* = 45°. It was manufactured using a Raise3D Pro2 3D printer via fused filament fabrication (FFF) with TPU. Printing parameters included a nozzle temperature of 240 °C, printing-bed temperature of 70 °C, and a speed of 18 mm/s. For the visual model, graphite conductive adhesive was injected into the grooves on the bottom surface to form the circuit. Additional details on hinges, circuits, and reconfiguration are provided in the Supplementary Materials.

### Experimental setup

The experimental setup for the quasi-static uniaxial compression experiments in Fig. 3E and Movie S3 to measure the stiffness of the metamaterial is as follows. The horizontal test machine used has a stroke of 800 mm and a load cell of 50 N, as shown in Fig. S10A. The specimen was placed on the fixed plate and compressed by the load plate connected to the load cell. Polytetrafluoroethylene (PTFE) films were applied on the plates, and an even layer of lubricating oil was applied to minimize friction between the specimen and the plates. Displacement control was applied in the compression experiments, and the loading rate was chosen to be 2 mm/min to eliminate dynamic effects. The final compression displacement was selected to be 3.99 mm, corresponding to a 3% compression strain. Additional details about the hinge stiffness measurements, the quasi-static uniaxial compression experiments in the *y* direction, and the real-time stiffness visualization experiments are provided in Notes S4 to S6.

### Statistical analysis

The experimental results (compressive stiffness) in Fig. 3E were derived from the force–displacement curves (Fig. S10B) measured in quasi-static experiments, using a linear fitting method. Statistical analysis was performed using Microsoft Excel 2021 and MATLAB R2020b.

## Data Availability

The data that support the findings of this study are available from the corresponding author upon reasonable request.

## Supplementary Materials

Supplementary 1 Notes S1 to S6 Figs. S1 to S12 Tables S1 and S2 Movies S1 to S4 Supplementary Code Reference [54]
