# Supplementary material for: Design and Visualization of a Hierarchical Metamaterial with Tunable Stiffness
Source: Research (Wash D C). 2025 Oct 16;8:0874. doi: 10.34133/research.0874 (PMC12696698; doi:10.34133/research.0874)
Supplement: Supplementary 1 — Notes S1 to S6 Figs. S1 to S12 Tables S1 and S2 Movies S1 to S4 Supplementary Code Reference [54] [file research.0874.f1.zip › Revised Supplementary Material.docx]

Supplementary Material for

**Design and Visualization of a Hierarchical Metamaterial with Tunable Stiffness**

Kaili Xi^1^, Xiaoyi Jiang^1^, Dechen Zhao^1^, Guimin Chen^2^, Jiayao Ma^1,3,^*, and Yan Chen^1,3,^*

^1^ School of Mechanical Engineering, Tianjin University, Tianjin 300350, China

^2^ State Key Laboratory for Manufacturing Systems Engineering and Shaanxi Key Lab of Intelligent Robots, Xi'an Jiaotong University, Xi'an, China

^3^ Key Laboratory of Mechanism Theory and Equipment Design of Ministry of Education, Tianjin University, Tianjin 300350, China

^*^Corresponding authors: Jiayao Ma: [jiayao.ma@tju.edu.cn](mailto:jiayao.ma@tju.edu.cn); Yan Chen: [yan_chen@tju.edu.cn](mailto:yan_chen@tju.edu.cn)

Kaili Xi and Xiaoyi Jiang are the co-ﬁrst authors.

**This PDF file includes:**

Notes S1 to S6

Figs. S1 to S12

Tables S1 to S2

Movies S1 to S4

**Note S1. Kinematic Analysis of the Metamaterials**

The metamaterial unit, composed of four isosceles right-angled triangular blocks with waist length *a* and thickness *t*, is connected by four hinges (black arcs) to form a planar four-bar linkage. As detailed in the main text, this unit exhibits a single DOF and bifurcates at *α*=180° and *β*=0°, providing two distinct motion paths. Figure S1A illustrates the complete motion paths of the unit, including both path 1 (a planar four-bar linkage) and path 2 (a two-bar linkage). The dimensions of the unit on each path are defined by its four side lengths *l*_1_, *l*_2_, *l*_3_, and *l*_4_ (Fig. S1B). On path 1, all hinges are active, and the unit behaves as a planar four-bar linkage, yielding the following relationship:

$l_{1}=l_{3}=2\sqrt{2}a\cos\left(\frac{90^{\circ}-\alpha}{2}\right)$, (S1)

$l_{2}=l_{4}=2a\sin\left(\frac{\alpha}{2}\right)$. (S1)

On path 2, the unit is reconfigured from the planar four-bar linkage to a two-bar linkage with only two active hinges. Equations (S1) and (S2) can still be used to calculate *l*_1_, *l*_2_, and *l*_4_, but *l*_3_ becomes

$l_{3}=2\sqrt{2}a\sin\left(\frac{\alpha-90^{\circ}}{2}\right)$. (S3)

Figure S1C to E illustrates the dimension of the unit with respect to *α*. Each path could be divided into two branches where all the four lengths vary monotonically with *α*, marked with 1A (90°~180°) and 1B (0°~90°) separated by point (*α*=90°, *β*=90°), and 2A (90°~180°) and 2B (180°~270°) separated by the bifurcation point (*α*=180°, *β*=0°). On each branch, the configuration of the unit is uniquely determined by any of its lengths except at the separation points.

When two units (denoted as unit 1 and unit 2) are connected by two new hinges (red arcs) to form a 1×2 metamaterial (Fig. S2), the geometric compatibility condition requires that the side length *l*_3_ of unit 1 is equal to the side length *l*_1_ of unit 2. According to Fig. S1C to E, if unit 1 is on branch 1A, 1B, or 2B, a corresponding unit 2 that satisfies this condition can be found on branch 1A, 2A, or 1B, respectively, resulting in nine possible combinations 1A&1A, 1A&2A, 1A&1B, 1B&IA, 1B&2A, 1B&1B, 2B&1A, 2B&2A, and 2B&1B. If unit 1 is on branch 2A, the only possible combination is 2A&1B. All ten possible combinations are presented in Fig. S2. Among these combinations, 1A&1A and 1A&2A have been employed to establish hierarchical levels in the metamaterial (Fig. S2A). In contrast, combinations that are not closely related to hierarchical levels, despite having rectangular connection quadrilaterals and the potential for further expansion (Fig. S2B), are not the focus of this study. Additionally, combinations with physical interference (1B&1B) and those with isosceles trapezoid connection quadrilaterals, which cannot be tessellated to form larger metamaterials with regular shapes (see Fig. S2C, are not considered. The reconfiguration process among all physically possible configurations is illustrated in Movie S1.

Subsequently, the kinematics of the 1×2 metamaterial is further analyzed by defining the kinematic variables *α*_1_, *β*_1_, *α*_1-2_, *β*_1-2_, *α*_2_, and *β*_2_, as shown in Fig. S3(a). The 1×2 metamaterial has four distinct motion paths (I-IV) and three bifurcation points. Starting from the 1A&1A combination on path I (*α*_1_=*α*_1-2_ + 90°), the two metamaterial units maintain identical configurations with all 10 hinges active. Here, the triangular blocks can be regarded as rigid links, while the hinges can be regarded as rotating joints. Using Chebychev's formula [54] for planar mechanisms, the degrees of freedom of the metamaterial, *M*, can be calculated as follows:

$M=3\left(n_{L}-1\right)-2n_{J}$. (S4)

Here, *n*_L_ represents the total number of links (7 mobile links and 1 fixed base), and *n*_J_ denotes the number of revolute joints (10 in this configuration). This calculation confirms that the configuration has a single DOF. The continuous motion persists until reaching the first bifurcation point (*α*_1_=90°, *α*_1-2_=0°), where the four adjacent blocks of the two units form two squares. This reconfiguration reduces the number of active hinges to 8, maintaining a single DOF with *M*=3(6-1)-2×7=1, where *n*_L_=6 and *n*_J_=7 (two contacting active hinges are considered as one revolute joint). The metamaterial then switches from path I to path II, where 0°≤*α*_1_≤180° and *α*_1-2_*=*0°. At the second bifurcation point (*α*_1_=180°, *α*_1-2_=90°), the eight basic triangular blocks merge into four larger ones, reaching the single-level configuration with one level 1 unit (6 active hinges). In this configuration, the metamaterial behaves as a planar four-bar linkage with a single DOF. The kinematics along paths III and IV, passing through the bifurcation point (*α*_1_=90°, *α*_1-2_=180°), are identical to those of the single unit shown in Fig. S1A. Thus, the 1×2 metamaterial maintains a single DOF except at the bifurcation points.

Moreover, using the same kinematic analysis approach, we find that the 2**×**2 metamaterial also exhibits a single DOF except at the bifurcation points. Using the recursive method, we can prove that by connecting quadrilaterals and adding two more hinges when the number of units is doubled, the resulting 1×2 and 2×2 metamaterials maintain their single DOF along the entire motion path, except at the bifurcation points. To validate this conclusion, we consider a representative configuration of the 4×4 metamaterial (Fig. S3B). This multi-level configuration, comprising two level 0 units, one level 1 unit, one level 2 unit, and one level 3 unit, can be translated into a planar mechanism with *n*_L_=20 links (19 mobile links and 1 fixed base) and *n*_J_=28 revolute joints. According to Chebychev's formula, *M*=3(*n*_L_-1)-2*n*_J_=1, confirming a single DOF (refer to Eqn. (S4)).

More generally, for a $2^{\left\lfloor i/2 \right\rfloor}\times2^{\left\lfloor\left( i+1 \right)/2 \right\rfloor}$ metamaterial with 2*^i^* level 0 units, reconfigured from a single-level configuration with only level 0 units into an arbitrary configuration with *U* units at different levels, it exhibits *n*_L_=4*U* links and *n*_J_=6*U*-2 revolute joints. Using Chebychev's formula, *M*=3(*n*_L_ -1)-2*n*_J_=1, ensuring a single DOF along the entire motion path, except at the bifurcation points. Following this construction method, a $2^{\left\lfloor i/2 \right\rfloor}\times2^{\left\lfloor\left( i+1 \right)/2 \right\rfloor}$ metamaterial with 2*^i^* units, which is derived by adding two hinges for each additional unit, has a total of *N*_T_=3×2*^i^*^+1^−2 hinges. And when it is reconfigured into a single-level configuration at level *i*, it has 6 active hinges when *i*≥1 (Fig. 1B and C).

**Note S2. Quantitative Analysis of the Number of Reconfigurable Configurations**

The following analysis quantitatively examines how reconfigurable configurations increase with the number of units, offering a detailed, recursive perspective (see Fig. S4). For a 1×1 metamaterial, there is only one single-level configuration (at level 0). For a 1×2 metamaterial, two single-level configurations exist: one with two level 0 units and another with one level 1 unit (Fig. S4). Subsequently, these two configurations combine to form the configurations for the 2×2 metamaterial, resulting in 2^2^=4 configurations. Including the single-level configuration at level 2, the 2×2 metamaterial has 5 configurations (3 single-level and 2 multi-level).

For the 2×4 metamaterial, the three single-level and two multi-level configurations of the 2×2 metamaterial can be combined to form 5^2^=25 configurations. Adding the single-level configuration at level 3, the 2×4 metamaterial has 26 configurations, including four single-level configurations (at levels 0, 1, 2, and 3, respectively) and 22 multi-level configurations. Extending this analysis to the 4×4 metamaterial, we find that it has 26^2^+1=677 configurations, among which 81 are multi-level ones.

In summary, for a $2^{\left\lfloor i/2 \right\rfloor}\times2^{\left\lfloor\left( i+1 \right)/2 \right\rfloor}$ metamaterial with 2*^i^* level 0 units, the number of configurations is given by (*P_i_*_-1_+*i*)^2^+1, where *P_i_*_-1_ is the number of multi-level configurations and *i* is the number of single-level configurations for the previous metamaterial size ($2^{\left\lfloor(i-1)/2 \right\rfloor}\times2^{\left\lfloor i/2 \right\rfloor}$), as described in the main text.

In addition, a symmetry analysis has been performed to identify symmetry-unique configurations and to develop an automated screening algorithm in MATLAB. Given the rectangular geometry of our metamaterial, only three types of symmetries need to be considered: second-order rotational symmetry, vertical mirroring, and horizontal mirroring. For the $2^{\left\lfloor i/2 \right\rfloor}\times2^{\left\lfloor\left( i+1 \right)/2 \right\rfloor}$ metamaterial with 2*^i^* level 0 units, there are *i*+1 single-level configurations, each of which is symmetry-unique due to the different hierarchical levels of the units. Therefore, only multi-level configurations require screening. Although it is possible to generate all multi-level configurations and then filter out the symmetry-unique ones, this approach is inefficient and memory-intensive.

Given this, we propose a recursive method to generate multi-level configurations, which eliminates a large number of symmetry-equivalent configurations upfront. Starting from a 2×2 metamaterial, which has three single-level configurations (at levels 0, 1, and 2, respectively), and one symmetry-unique multi-level configuration (Fig. 2A). Through three symmetry operations (second-order rotational symmetry, vertical mirroring, and horizontal mirroring), this symmetry-unique multi-level configuration generates only one additional symmetry-equivalent configuration (the grey one in Fig. 2A). Then these configurations can be combined to construct multi-level configurations for the 2×4 metamaterial, which can be divided into four groups:

1. Pairwise combinations of single-level configurations yield three symmetry-unique multi-level configurations (Fig. S5A).
2. Self-combination of the symmetry-unique multi-level configuration results in another symmetry-unique configuration (Fig. S5B).
3. Combinations of single-level and multi-level configurations produce six additional configurations, of which only the top three are symmetry-unique (Fig. S5C).
4. Pairwise combinations of multi-level configurations provide one more symmetry-unique configuration (Fig. S5D).

Thus, the 2×4 metamaterial has a total of eight symmetry-unique multi-level configurations. In groups 3 and 4, symmetry-equivalent multi-level configurations are included because combining them can produce new symmetry-unique configurations in larger metamaterials.

For the 4×4 metamaterial, we first generate all symmetry-equivalent configurations of the eight multi-level configurations of the 2×4 metamaterial using the three symmetry operations (Fig. S5E), and then apply the same methods to generate and screen symmetry-unique multi-level configurations. This approach reduces the number of configurations to be screened: from 22 to 11 for the 2×4 metamaterial, and from 672 to 86 for the 4×4 metamaterial.

To facilitate this process, we introduce a labeling method for each configuration. For example, a typical 2×4 metamaterial configuration at levels 0, 1, and 2 and its three symmetry-equivalent configurations can be represented using the 2×4 table. The table assigns values based on the hierarchical levels and positions of the units: level 0 units occupy one cell with a value of 0, level 1 units occupy two adjacent cells in the same row with a value of 1, and level 2 units occupy a 2×2 area with a value of 2. This labeling method allows us to develop an automated screening algorithm in MATLAB that efficiently generates and combines configurations while identifying symmetry-unique ones, see Supplementary Code for details.

**Note S3. Theoretical Modeling of Tunable Stiffness in Metamaterials**

This Note presents a theoretical model for the tunable stiffness of a general $2^{\left\lfloor i/2 \right\rfloor}\times2^{\left\lfloor\left( i+1 \right)/2 \right\rfloor}$ metamaterial constructed from 2*^i^* level 0 units, which has a total of *N*_T_=3×2*^i^*^+1^−2 hinges (Note S1, Supplementary Material). Since the strain energy of the metamaterial can be calculated by summing the strain energy of the active hinges, as described in the main text, we first explore the number of active hinges at different configurations. When all units are at level 0, the number of active hinges is equal to the total number of hinges, 3×2*^i^*^+1^−2. In general, a level *j* unit (*j*≥1) has a total of *n_j_*_,T_ =3×2*^j^*^+1^−2 hinges, but only six of them are active. For example, a level 1 unit has 10 hinges, but only six are active (Fig. 1B). Thus, a level *j* unit contributes *n_j_*_,inact_=3×2*^i^*^+1^−8 inactive hinges. Denote the number of level j units as *u_j_*_​_. When *j*=1, *u*_1_≤2*^i^*^-1^ because reconfiguring all 2*^i^* level 0 units into level 1 units generates only 2*^i^*^-1^ level 1 units. Similarly, *u*_2_≤2*^i^*^-2^, as reconfiguring all units into level 2 produces 2*^i^*^-2^ level 2 units. Generally, the number of level *j* units *u_j_* varies from 0 to 2*^i^*^-^*^j^*. Therefore, when the metamaterial is reconfigured from the single-level configuration with only level 0 units into a configuration composed of *u_j_*_​_ level *j* units (*j* varies from 0 to *i*), the number of active hinges *N* can be calculated by subtracting the inactive hinges contributed by each level *j* unit (*j*≥1) (Equation (3)).

Subsequently, the compressive stiffness of the metamaterial along both the *x*-axis and *y*-axis are analyzed. Defining the angle between the left top block and the vertical direction as *θ*/2, the angles of hinges for the metamaterials with even-numbered-level and odd-numbered-level blocks could be derived geometrically, as illustrated in Fig. S6A and B. The angles of six group hinges (#1~#6) are 90°-*θ*, *θ*, 90°-*θ*, *θ+*90°, 180°-*θ*, *θ*, respectively. Therefore, the conclusion in the main text for a 2×2 metamaterial can be extended to this general metamaterial, i.e., all the hinges rotate by the same magnitude *△θ*. Consequently, the strain energy of the metamaterial can be calculated as

$U=\frac{1}{2}\left(\Delta\theta\right)^{2}\sum_{m=1}^{N} k_{m}$, (S5)

where *k*_m_ (*m*=1, 2, …, *N*) represents the rotational stiffness of the *m*th hinge, and *N* is the number of active hinges.

For the *x*-axis direction, the axial force exerted on the metamaterial can be obtained by differentiating the energy with respect to the width *W* of the metamaterial

$F=\frac{dU}{dW}=2^{-\left\lfloor\left(i+1\right)/2\right\rfloor}\frac{\text{Δ}\theta}{a\sin\left(\text{π}/4-\theta/2\right)}\sum k_{m}$, (S6)

in which the width *W* is

$W=2^{\left\lfloor\left(i+1\right)/2\right\rfloor+1}a\cos\left(\text{π}/4-\theta/2\right)$. (S7)

And the stiffness of the metamaterial can also be derived by further differentiating the force with respect to the width

$K=\frac{dF}{dW}=2^{-\left(2\left\lfloor\left(i+1\right)/2\right\rfloor+1\right)}\sum k_{m}\frac{\left(2+\cot\left(\text{π}/4-\theta/2\right)\text{Δ}\theta\right)}{a^{2}\sin^{2}\left(\text{π}/4-\theta/2\right)}$. (S8)

When all the hinges have identical rotational stiffness *k*, Eqns. (S6) and (S8) can be rewritten as

$F=2^{-\left\lfloor\left(i+1\right)/2\right\rfloor}\frac{\text{Δ}\theta}{a\sin\left(\text{π}/4-\theta/2\right)}Nk$. (S9)

$K=2^{-\left(2\left\lfloor\left(i+1\right)/2\right\rfloor+1\right)}\frac{\left(2+\cot\left(\text{π}/4-\theta/2\right)\text{Δ}\theta\right)}{a^{2}\sin^{2}\left(\text{π}/4-\theta/2\right)}Nk$. (S10)

Therefore, the force and stiffness in the *x* direction of the metamaterial have a linear relationship with the number of active hinges.

In addition, the compressive stiffness of the metamaterial in the *y* direction can be obtained by following the same procedure. The stiffness can be obtained by taking the second derivative of the strain energy with respect to the height *H* of the metamaterial

$K_{H}=\frac{d^{2}U}{dH^{2}}=2^{-\left(2\left\lfloor\left(i+1\right)/2\right\rfloor+2\right)}\frac{\left(2-\cot\left(\theta/2\right)\text{Δ}\theta\right)}{a^{2}\sin^{2}\left(\theta/2\right)}Nk$. (S11)

where the height *H* can be calculated as

$H=2^{\left\lfloor\left(i+1\right)/2\right\rfloor+1}\sqrt{2}a\cos\left(\frac{\theta}{2}\right)$. (S12)

Thus, the stiffness in the *y* direction of the metamaterial also shows a linear relationship with the number of active hinges.

**Note S4. Manufacture of Physical Specimens**

For the metamaterial with all 94 hinges active in Fig. S7A, the triangular blocks have side length *a*=18mm, thickness (perpendicular to the metamaterial surface) *t*=20mm, and the metamaterial’s initial configuration is represented with the initial angle *θ*_0_=45°. Four types of flexible hinges, noted as #1, #2, #3, and #4, were designed, as shown in Fig. S7B. All hinges are rectangular with identical width *w*=0.6mm, see Table S1 for their specific parameters. The 3D printing technique was adopted to manufacture the metamaterial with thermoplastic polyurethanes (TPU) using a 3D fused filament fabrication (FFF) printer (Raise3D Pro2). During the printing process, the nozzle temperature was 240°C, the printing-bed temperature was 70°C, and the printing speed was 18mm/s. In addition, the extrusion line width of the nozzle was adjusted to 0.3mm so that there were two layers of wires in the width direction of the hinges. One of the fabricated specimens is presented in Fig. 3D as an example. Specimens with 74, 62, 30, 14, and 6 active hinges were obtained by reconfiguration from those with 94 active hinges through external force and heat treatment, as shown in Fig. 3D. Five new flexible hinges in the reconfigured configurations are numbered #5, #6, #7, #8, and #9, respectively.

Specifically, we adjusted the thickness of hinges #1~#4 to compensate for the stiffness deviation caused by 3D printing, achieving uniform rotational stiffness among these four types of hinges, as the rotational stiffness of the straight-beam hinge is directly proportional to its thickness (perpendicular to the metamaterial surface). In addition, to avoid the effects of physical contact during reconfiguration, we place the hinges that will merge to form new hinges in separate locations: the upper half (*t*=10~20 mm) and the lower half (*t*=0~10 mm) of the metamaterial. For example, as shown in Fig. S7A, the red-marked hinge #1 is located in the upper half, while its corresponding grey-marked hinge #1*'* is located in the lower half. These two hinges have identical parameters. This is experimentally verified in the following Note, where the rotational stiffness of hinge #6 is twice that of hinge #4, with similar relationships observed for hinges #7 and #5, and #9 and #8.

Moreover, the reconfiguration was achieved through a three-step process. First, the specimen was manually deformed to the target configuration at room temperature and then placed in a mold that matched the shape of the specimen. Second, the specimen and the mold were put into a heating furnace at 130°C for 2 hours. Finally, the specimen was cooled down at room temperature for 1 hour and removed from the mold. After the process, the target configuration could be well preserved without external force. The reconfiguration does not affect the hinge stiffness, as confirmed experimentally in the following subsection.

**Note S5. Stiffness of Hinges and Metamaterials**

Firstly, to obtain the rotational stiffness of the flexible hinges in the six configurations of the metamaterials in Fig. 3D of the main paper, a loading structure as shown in Fig. S8A was designed, in which the flexible hinge was connected to two rigid blocks. An external force *F* along the *z*-axis was applied to the PE line, and the pins connected to the rigid blocks were pulled through the PE (polyethylene) line to cause the flexible hinge to bend, producing a rotation angle *θ*. Therefore, the rotational stiffness of the flexible hinge is

$k=\frac{M}{\theta}=\frac{FL}{\theta_{\text{Top}}+\theta_{\text{Bottom}}}$, (S13)

where *F* is the external force applied to the rigid blocks along the *z*-axis; *L* is the length between the point where the external force is applied and the center point of the hinge along the *x*-axis; *θ*_Top_ and *θ*_Bottom_ are the rotation angles of the upper and lower parts of the flexible hinge, respectively.

As shown in Fig. 3D, there are nine flexible hinges in the six tested configurations of the 4×4 metamaterial, which are numbered #1~#9. Among them, hinges #1~#4 were individual hinges obtained directly by 3D printing, so the loading structures were designed directly based on the original metamaterials. Hinges #5 and #8 are obtained by reconfiguration through external force and heat treatment, so they are first designed, and then deformed and heat-treated in accordance with the configuration conversion procedure of the metamaterials. Hinges #6, #7, and #9 are formed by two hinges from hinges #5, #4, and #8, respectively. These hinges were also obtained from hinges #5, #4, and #8 with reconfiguration under heat treatment. To ensure the consistency of the flexible hinges in the measuring structure with those in the metamaterial, the same 3D printing settings for the metamaterials were adopted. Figure S8B shows the 3D printed loading structures for hinges #1~#9.

Quasi-static uniaxial tensile experiments were conducted to measure the rotational stiffness of these flexible hinges. An Instron 5982 testing machine with a load cell of 100N was utilized, and the experimental setup is illustrated in Fig. S8C. Displacement control was applied in the tensile experiments, and the loading rate was chosen as 1 mm/min to eliminate dynamic effects. The experimentally measured bending moment versus rotation angle is shown in Fig. S8D, and the corresponding rotational stiffness can be obtained by linear fitting.

To investigate the fatigue characteristics of TPU hinges after thermal reconfiguration, we measured the rotational stiffness of a hinge after repeated reconfiguration cycles. Taking hinge #2 as an example, we first measured its initial rotational stiffness in its printed state. Then, we subjected it to cyclic reconfiguration, switching between hinges #2 and #5 (each reconfiguration process representing one round). As shown in Fig. S9, the initial rotational stiffness of hinge #2 was 9.034 N·mm/rad. Over the first few rounds, the rotational stiffness decreased to approximately 6.829 N·mm/rad and stabilized after 14 rounds. This indicates that the TPU hinges exhibit consistent performance after repeated reconfiguration rounds (excluding the first 12 rounds), demonstrating good resistance to fatigue under the tested conditions and enabling multiple reuses.

The rotational stiffness of the nine flexible hinges tested after 14 rounds of thermal reconfigurations is summarized in Table S2. These results confirm that the rotational stiffness of hinges #1~#5 and #8 remains consistent. Hinge #6 has twice the rotational stiffness of hinge #4, and similar relationships are observed between hinges #7 and #5, as well as between hinges #9 and #8. The rotational stiffness (*k*) of each hinge used in this paper is the average value of the basic hinge types #1~#4, which is 6.776 N·mm/rad.

Subsequently, the compressive force-displacement relationship of the metamaterial in the *x* direction can be calculated using Equation (S9). Here, the angle *θ* varies from 45° to 37.29°, and *△θ* varies from 0° to 7.71°, corresponding to a 3% strain. The theoretical compressive stiffness of the 4×4 metamaterial in different configurations can be obtained by linear fitting, as illustrated in Fig. 3E and Fig. S10E.

To validate these theoretical results, quasi-static uniaxial compression tests were conducted on 4×4 metamaterials. The experimental setup, shown in Fig. S10A, includes a horizontal test machine with an 800 mm stroke and a 50 N load cell. The specimen was placed on a fixed plate and compressed by the load plate connected to the load cell. PTFE films were applied to the plates, and an even layer of lubricating oil was applied to minimize friction between the specimen and the plates. Compression tests were conducted under displacement control at a rate of 2 mm/min to eliminate dynamic effects, with a final compression displacement of 3.99 mm, corresponding to a 3% strain. Experimental force-displacement results for the 4×4 metamaterial in six configurations (Fig. 3D) and in four configurations with 54 active hinges (Fig. S10C) are presented in Fig. S10B and D, respectively. The compression stiffness values of the tested specimens, obtained by linear fitting, are shown in Fig. 3E and Fig. S10E.

Similarly, the compressive stiffness in the *y* direction was measured using a comparable setup (Fig. S10F), with the specimen compressed in the *y* direction by a 50 N load cell. The final compression displacement was 5.64 mm, corresponding to a 3% strain. The obtained compression stiffness values for the six configurations (with 94, 62, 54, 30, 14, and 6 active hinges, respectively) shown in Fig. 3D are presented in Fig. S10G.

**Note S6. Design, fabrication, and Validation of the 4×4 Specimen for Real-time Stiffness Visualization**

The logic diagram (Fig. S12A) of the 4×4 metamaterial comprises 23 pairs of buffer and NOT gates, with eight logic operators for *j*=0 (inputs *A*_0,_*_s_*, *s*=1 to 8), four for *j*=1 (inputs *A*_1,_*_s_*, *s*=1 to 4), two for *j*=2 (inputs *A*_2,_*_s_*, *s*=1 to 2) and one for *j*=3 (input *A*_3_). Based on this diagram, the global circuits (Fig. S12B) are constructed with outputs *Q*_1_-*Q*_22_ (circled numbers 1-22) controlling 22 pink LEDs and output *Q*_23_ (the circled number 23) controlling the yellow LED. Figure S12C shows the design of this specimen, where grooves are strategically reserved at locations corresponding to the navy circuits in Fig. S12B. For circuits along the outer edges that are directly connected to the power input (blue nodes), the grooves are connected by embedded conductive threads (orange arcs). Output nodes are indicated by corresponding-colored dots. Enameled wires (brown) for external device connections are pre-embedded at input and output node locations (Fig. S12D). Graphite conductive adhesive is injected into the grooves and allowed to cure for 24 hours at room temperature (Fig. S12E). Note that synchronized output pairs, such as nodes ⑨ and ⑩, are treated as a single node with a weight of 2, requiring only one enameled wire. Finally, the input node's wires are connected to the positive terminal of a 5V power supply, and the 16 output nodes' wires are connected to the chip's 16 inputs. The chip processes the output data and provides the results to the LED row and 7-segment display, indicating the number of active hinges *N* in real time.

The logic behind the chip's data processing is as follows:

1. Results for the LED row: The LED bar consists of 23 square LEDs, with the first LED for yellow and the rest for red. Initially, all the LEDs are lit. When a node with a weight of 1 is activated (the corresponding output *Qs*​=1), one pink LED at the end of the bar is switched off. When a node with a weight of 2 is activated (*Qs*​=1), two pink LEDs at the end of the bar are switched off.
2. Results to the 7-segment display: The displayed number is calculated using the formula *N*=6+4(*q*_1_-1)+8*q*_2_, where *q*_1_ is the number of nodes with a weight of 1 that remain inactive (*Qs*​=0), and *q*_2_​ is the number of nodes with a weight of 2 that remain inactive (*Qs*​=0).

**Figs. S1 to S12**
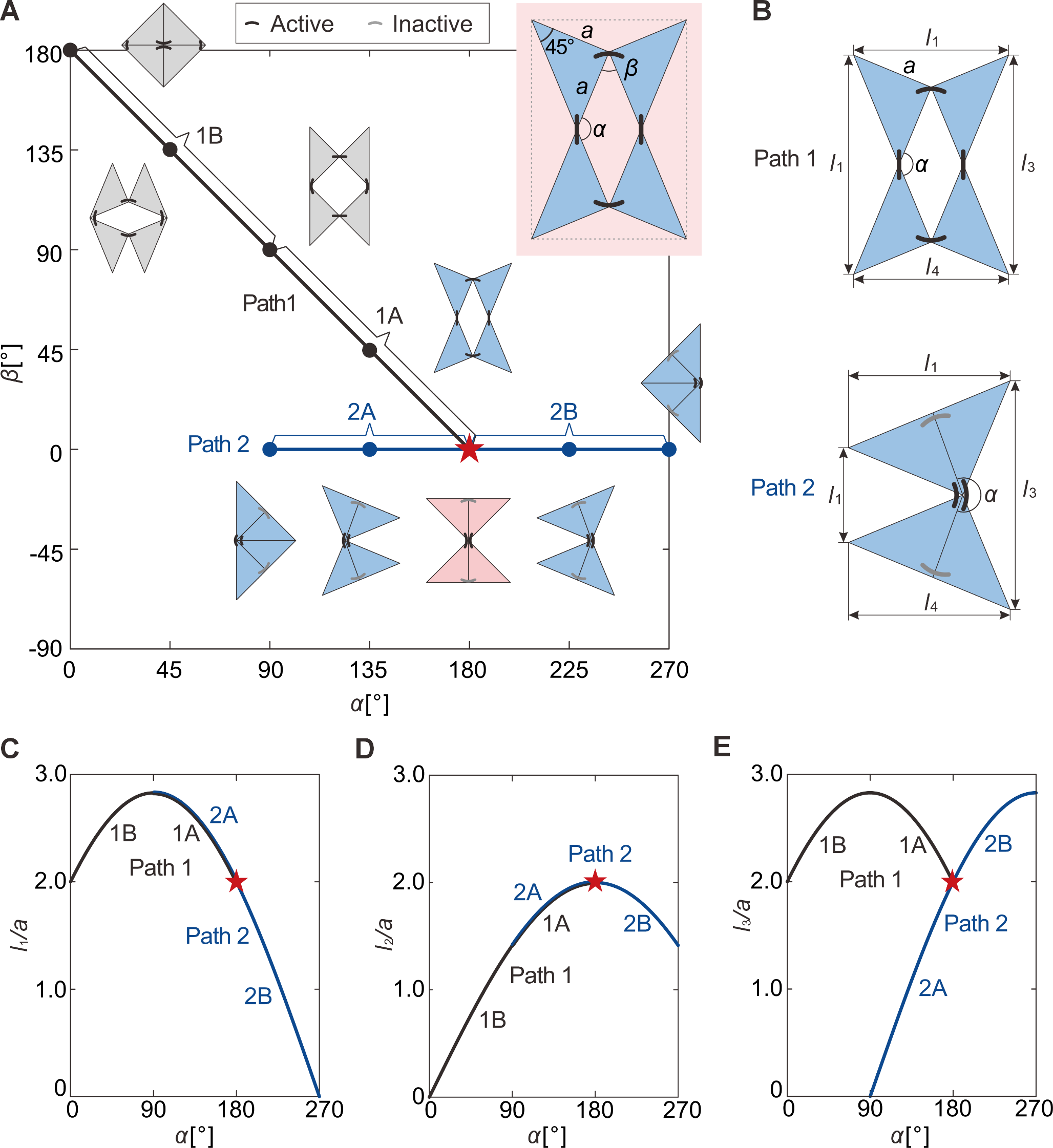


Fig. S1. Kinematic analysis of the single unit. (A) Unit cell and its two motion paths, with typical configurations on each path. Grey configurations are not considered in the main text. (B to E) Connection quadrilaterals on different motion path branches, with their (C) width, (D) left side length, and (E) right side length normalized with the triangles’ waist length a varying with *α*.
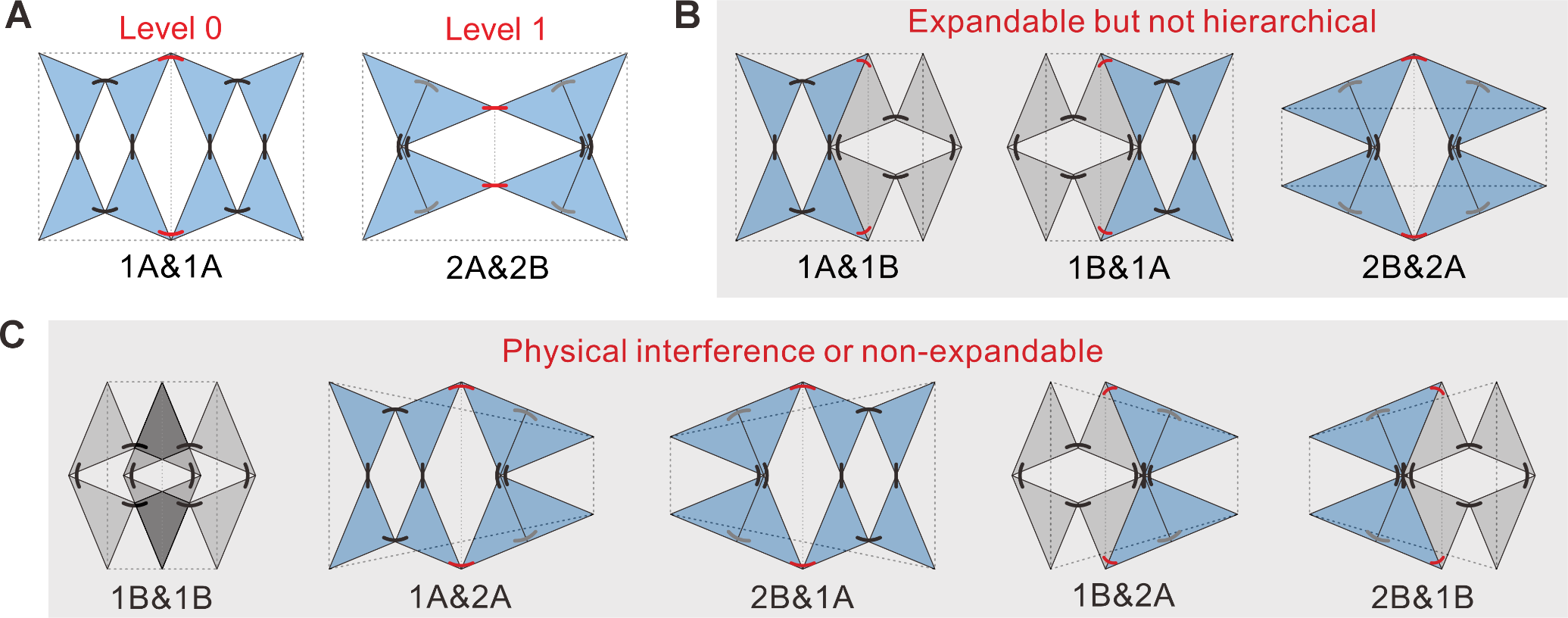


Fig. S2. Possible combinations of two units. (A) Hierarchical combinations. (B) Expandable but not hierarchical combinations. (C) Physical interference or non-expandable combinations.


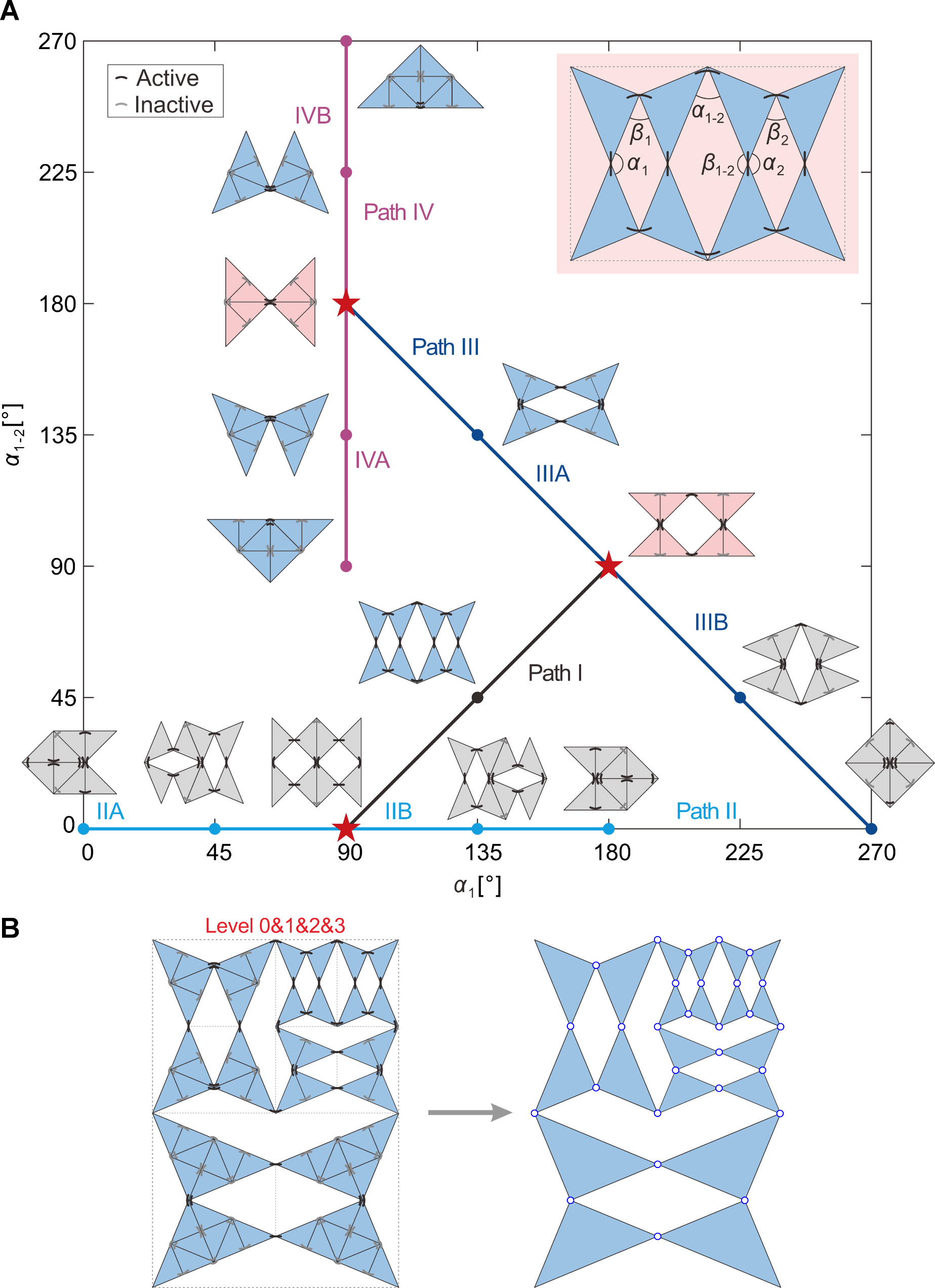


Fig. S3. Kinematic analysis of the metamaterials. (A) The kinematics of the 1×2 metamaterial, determined by two kinematic variables *α*_1_ and *α*_1-2_, with four motion paths and three bifurcation points (marked with red stars). Grey configurations are not considered in the main text. (B) A representative configuration of the 4×4 metamaterial at level 0&1&2&3 and its corresponding planar mechanism.


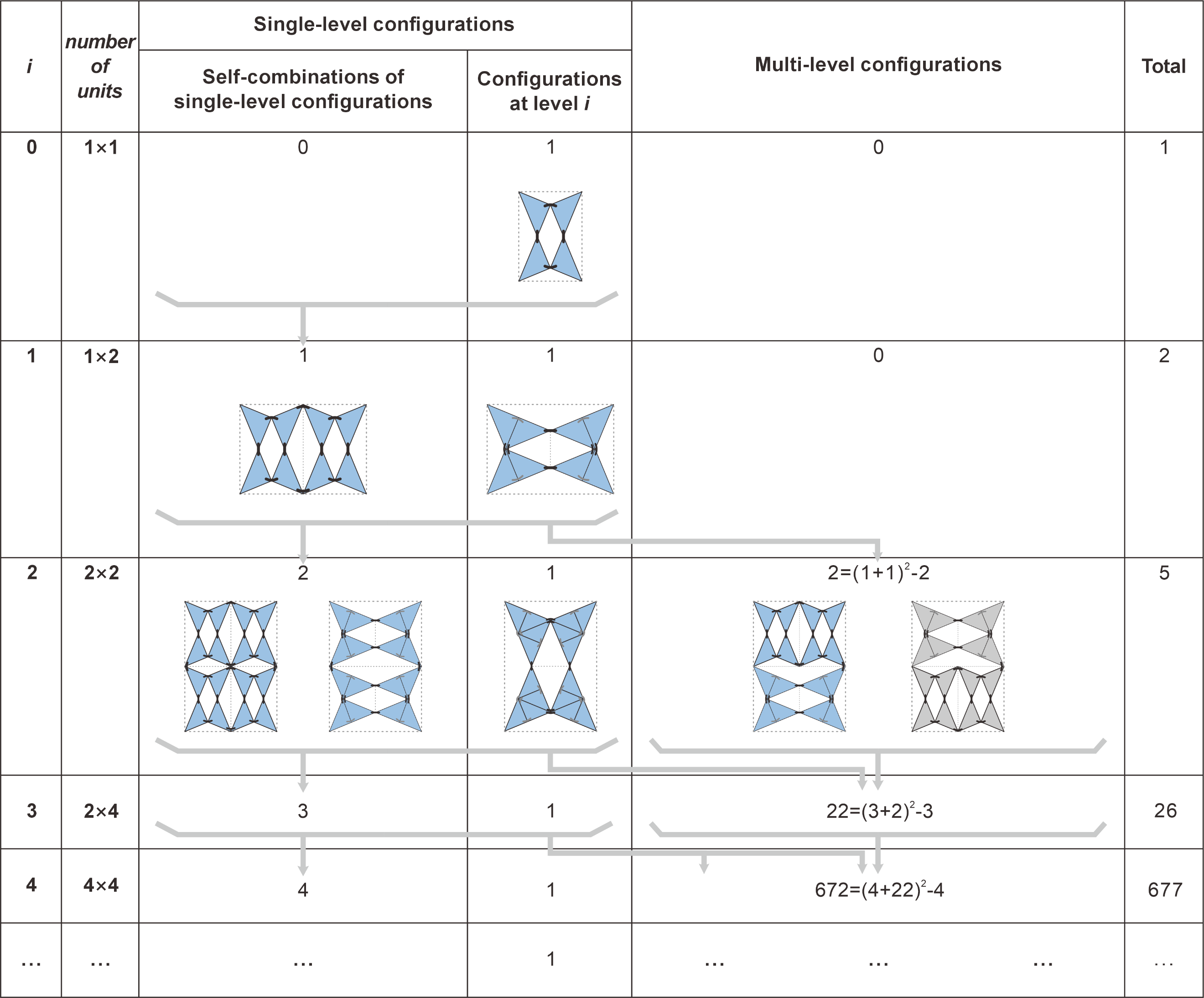


Fig. S4. Recursive analyses of the number of reconfigurable configurations. The two multi-level configurations of the 2×2 metamaterial are symmetry-equivalent, so we retain only one representative configuration (the blue one) to identify the symmetry-unique configurations.


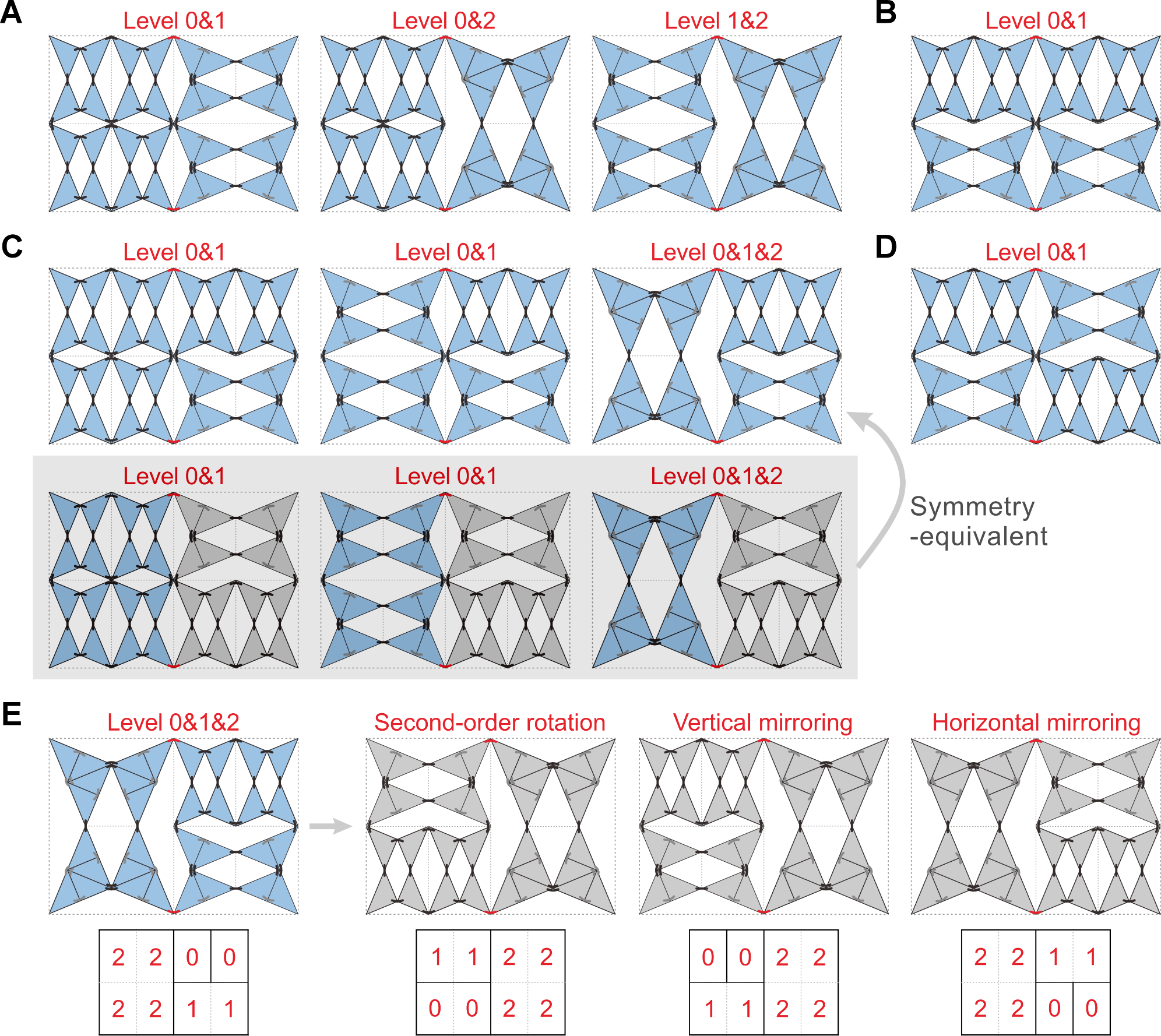


Fig. S5. Quantitative analysis of symmetry-unique configurations for the 2×4 metamaterial. (A) Pairwise combinations of the single-level configurations of the 2×2 metamaterial. (B) Self-combination of the symmetry-unique multi-level configuration of the 2×2 metamaterial. (C) Combinations of single-level configurations and the multi-level configurations (symmetry-unique and their equivalents) of the 2×2 metamaterial. The top three configurations are symmetry-unique, while the bottom three are symmetry-equivalent to the top three. (D) Pairwise combination of multi-level configurations (symmetry-unique and its equivalents) of the 2×2 metamaterial. (E) A representative configuration of the 2×4 metamaterial at level 0&1&2 and its three corresponding symmetry-equivalent configurations derived from symmetry operations (second-order rotational symmetry, vertical mirroring, and horizontal mirroring). These configurations can be represented in a 2×4 table, with values assigned based on the hierarchical levels and positions of the units within the configurations.


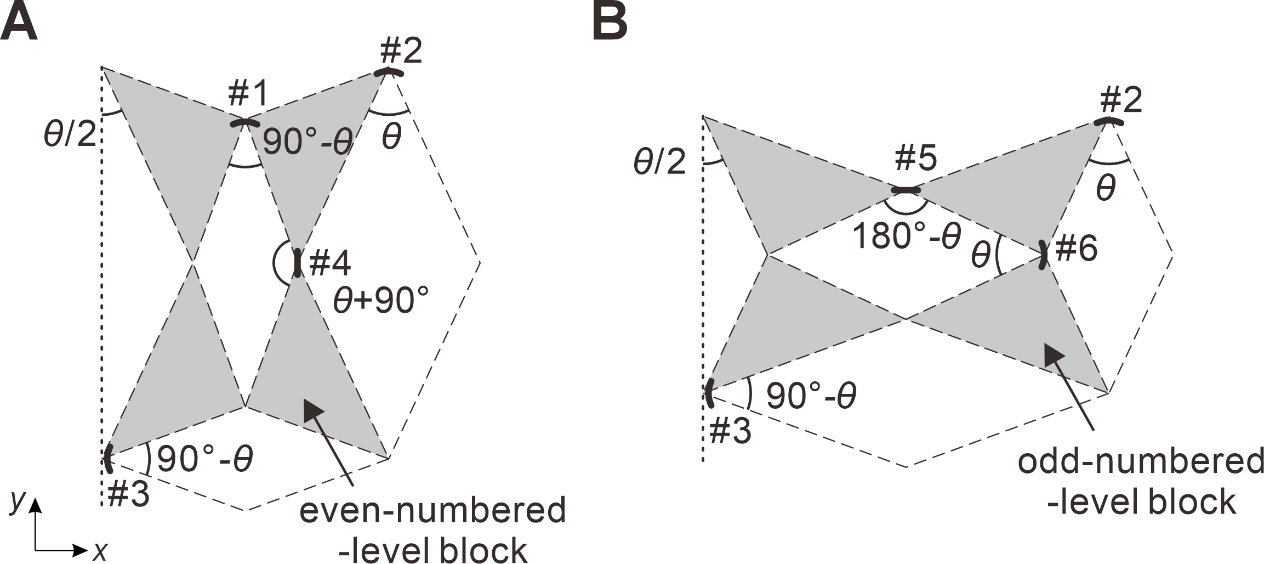


Fig. S6. Theoretical modeling of the metamaterial. Angles of different types of hinges related to (A) even-numbered-level blocks; (B) old-numbered-level blocks.


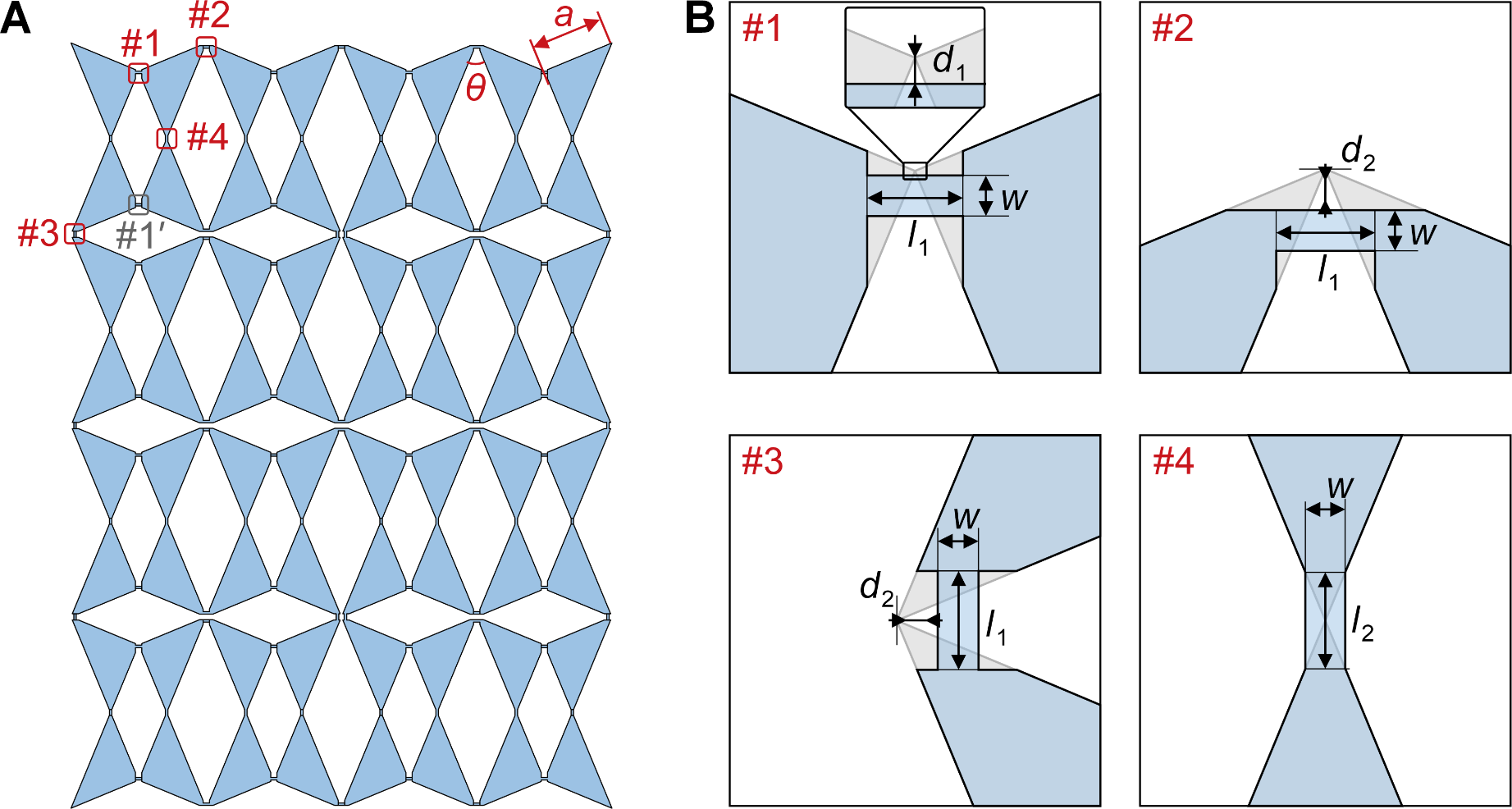


Fig. S7. Design details of 4×4 metamaterials. (A) Design of a 4×4 metamaterial with 94 active hinges. (B) Design of the four types of hinges numbered #1, #2, #3, and #4. Gray shadow and blue shadow represent the metamaterial before and after hinge design respectively.

**
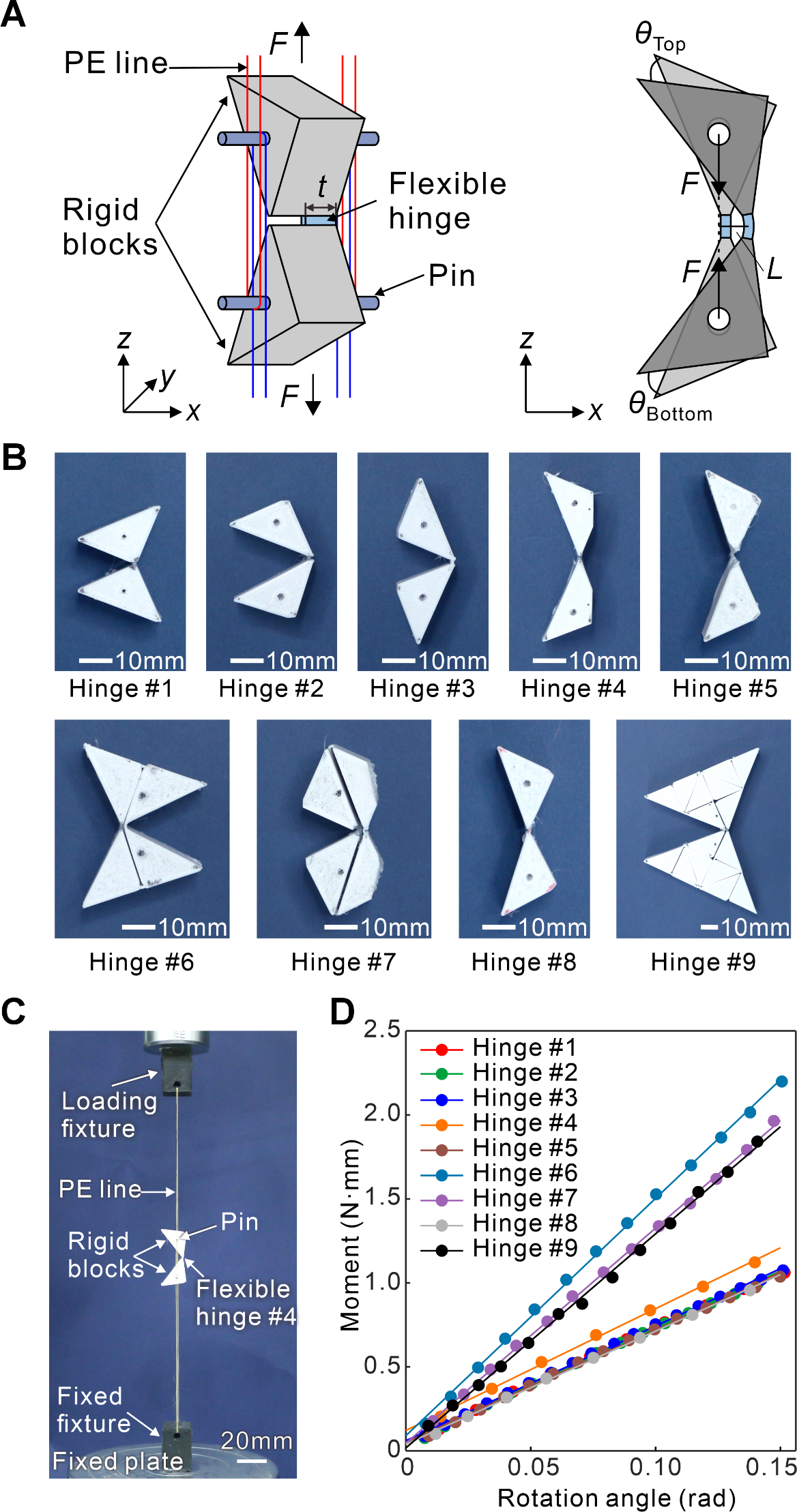
**

Fig. S8. The measurement of the rotational stiffness of flexible hinges. (A) Sketches of the loading structure and deformation mode of flexible hinges. (B) 3D printed loading structure corresponding to the nine flexible hinges. (C) Quasi-static axial tensile test setup of flexible hinges. (D) Experimental bending moment-rotation angle relationships of the nine flexible hinges marked with dots and the linear fitting results marked with solid lines. All experimental curves presented are the mean curves from three replicates.

**
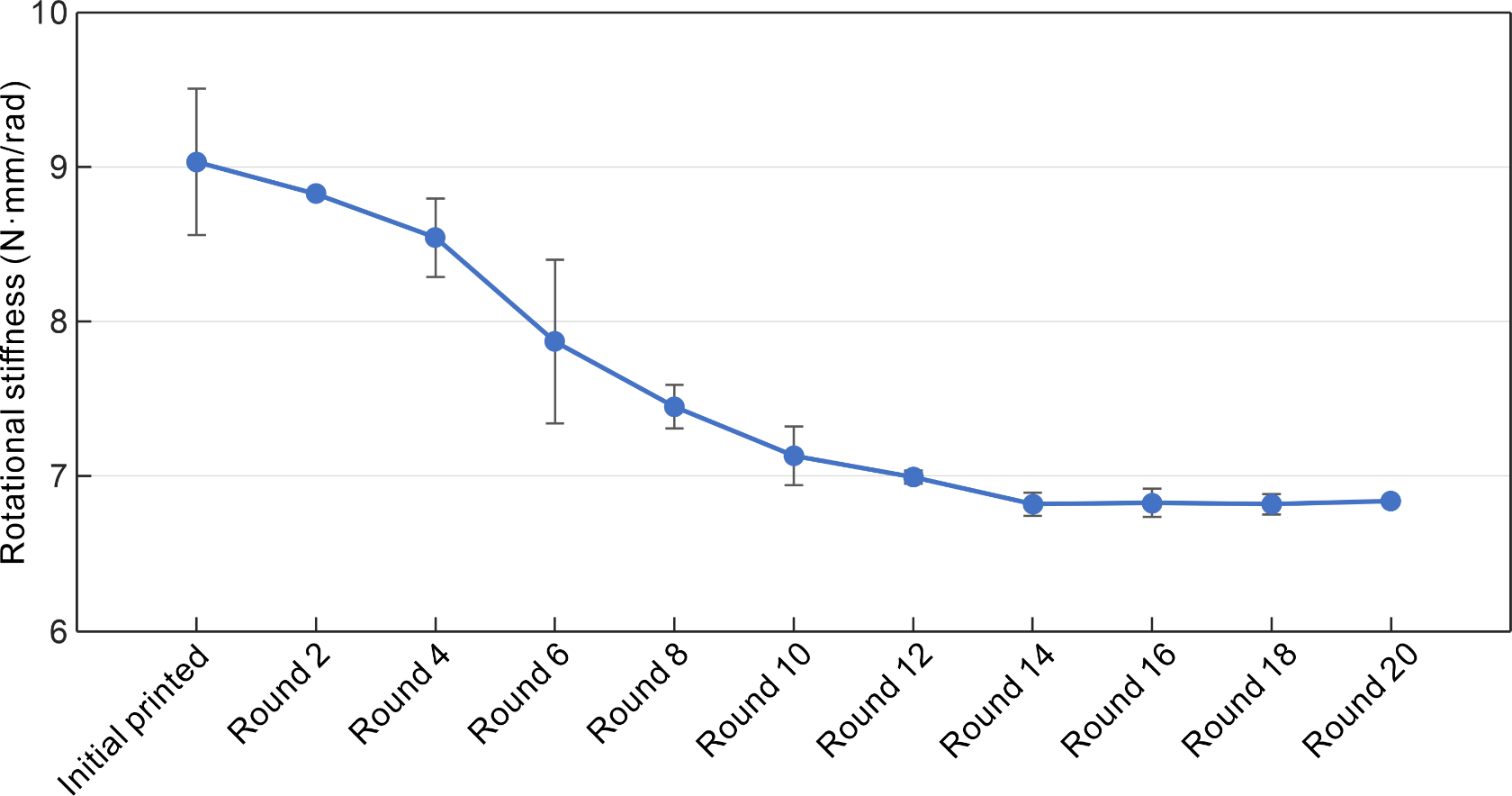
**

Fig. S9. Rotational stiffness of hinge #2 in its initial printed state and after 20 thermal treatment rounds (each round consisting of 2 hours at 130°C followed by 1 hour of cooling at room temperature). Error bars indicate standard deviation.

**
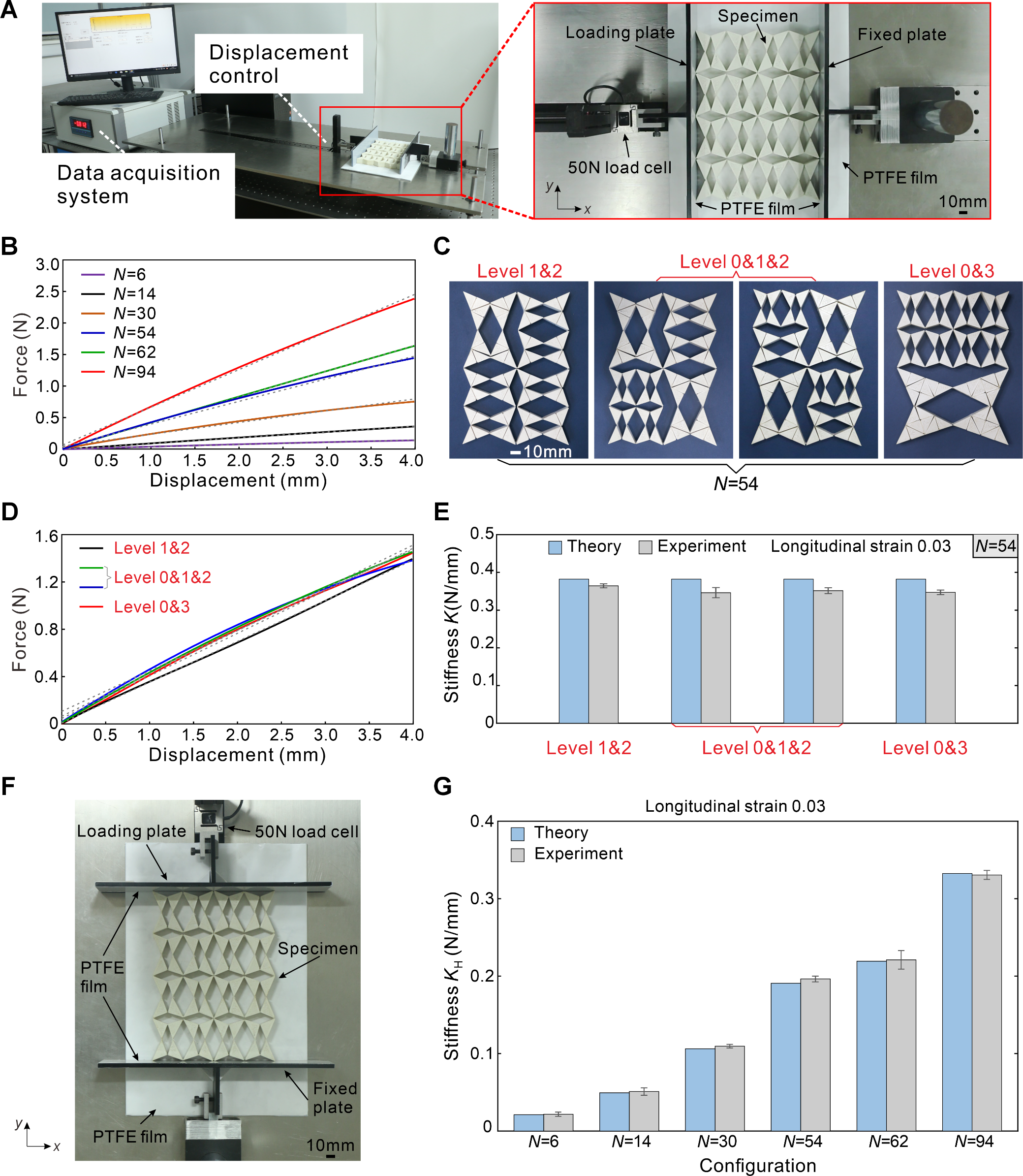
**

Fig. S10. Compressive stiffness characterization of 4×4 metamaterials. (A) Experimental setup for quasi-static axial compression in the *x* direction. (B) Experimental force-displacement curves for six configurations (corresponding to Fig. 3(d), with active hinge numbers *N*=6, 14, 30, 54, 62, and 94), along with their linear curve fitting results (dashed lines). All experimental curves presented are the mean curves from three replicates. (C) Specimens of the 4×4 metamaterial in four multi-level configurations (level 1&2, two different level 0&1&2, and level 0&3), each with 54 active hinges. (D) Experimental force-displacement curves of four multi-level configurations in (C), along with linear curve fitting results (dashed lines). (E) Comparison of theoretical and experimental compressive stiffness for four configurations in (D) under 3% strain. (F) Experimental setup for quasi-static axial compression in the *y* direction. (G) Comparison of theoretical and experimental compressive stiffness for six configurations (with 94, 62, 54, 30, 14, and 6 active hinges, respectively) in Fig. 3D under 3% strain in the *y* direction.


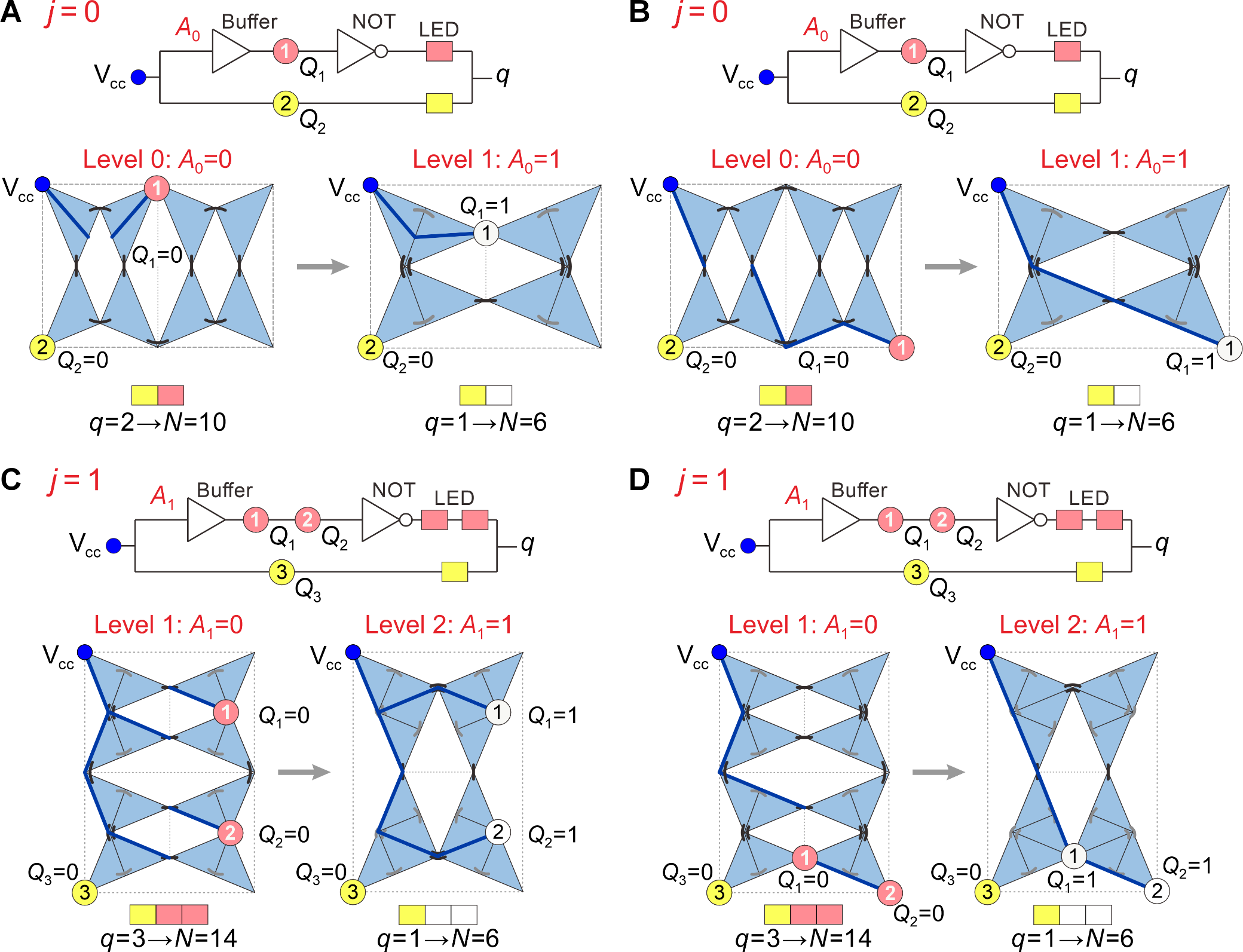


Fig. S11. Some feasible circuit arrangements for logic operators which describe the reconfiguration from two level *j* units to one level *j*+1 unit, with corresponding electrical outputs. (A, B) Two example arrangements for *j*=0 with inputs *A*_0_. (C, D) Two example arrangements for *j*=1 with inputs *A*_1_.


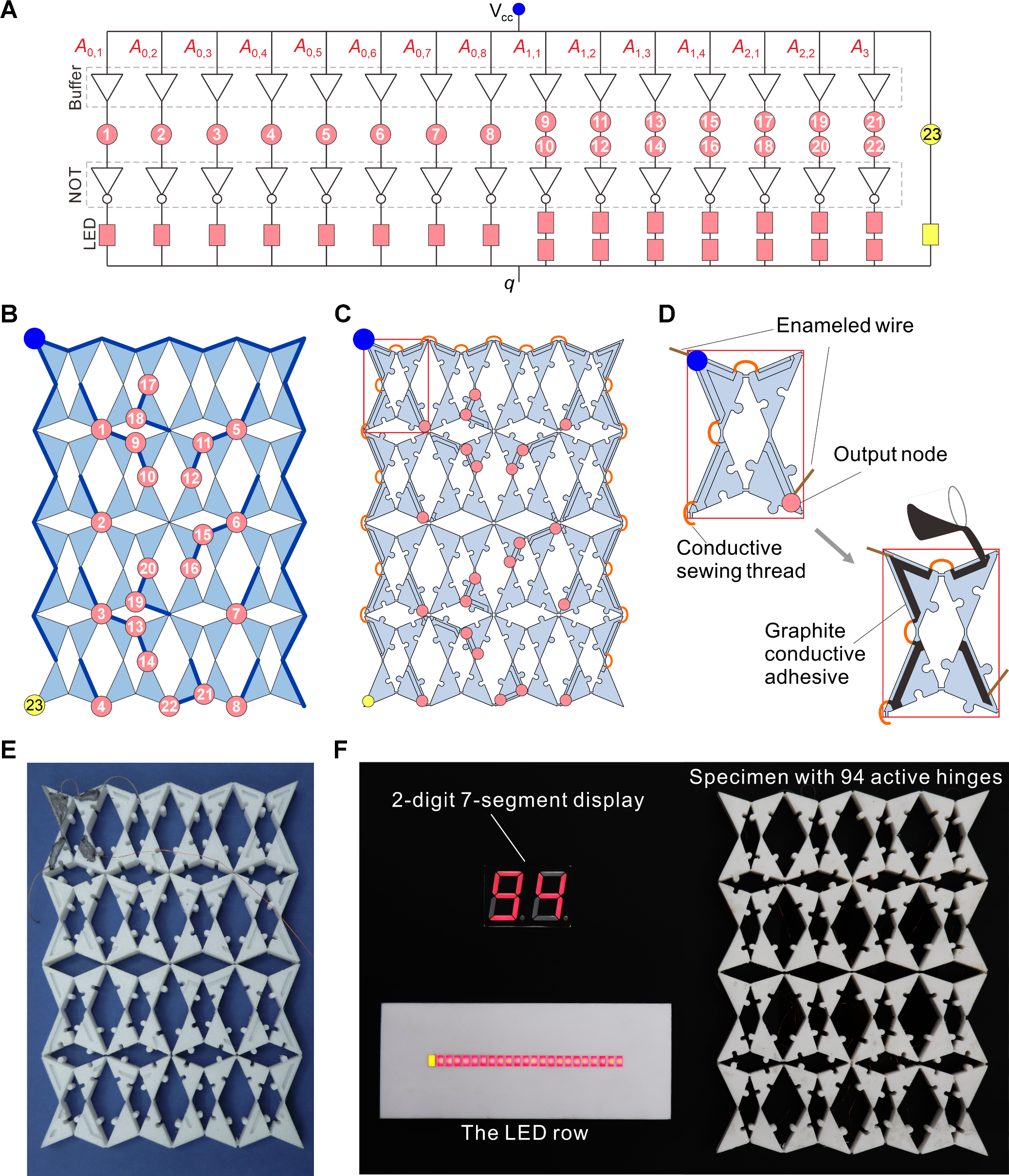


Fig. S12. Design and fabrication of the 4×4 specimen for real-time stiffness visualization. (A, B) Logic diagram and design schematic of the 4×4 metamaterial, yielding outputs *Q*_1_-*Q*_23_. The power input terminal is marked by a blue dot, and output nodes are marked by circled numbers. Circuits are represented by navy lines. (C) The design of the printed specimen, with grooves reserved at the positions corresponding to the navy circuit in (B), and the outer edge always maintaining a continuous circuit through the embedded conductive sewing threads. (D) Fabrication process. Input and output nodes are pre-embedded with enameled wires, followed by the injection of graphite conductive adhesive into the grooves at room temperature, and left to solidify for 24 hours. (E) The specimen in manufacturing. (F) The 4×4 metamaterial specimen at level 0 with 94 active hinges.

**Tables S1 to S2**

Table S1. Design parameters of hinges #1-#4 (mm).

| Hinge number | #1 | #2 | #3 | #4 |
| --- | --- | --- | --- | --- |
| Length | *l*_1_=1.55 | *l*_1_=1.55 | *l*_1_=1.55 | *l* _2_=1.45 |
| Width | *w*=0.60 | *w*=0.60 | *w*=0.60 | *w*=0.60 |
| Thickness (perpendicular to the metamaterial surface) | *t*_1_=5.60 | *t*_2_=4.85 | *t*_3_=5.30 | *t*_4_=9.05 |
| Offset distance | *d*_1_=0.06 | *d*_2_=0.68 | *d*_2_=0.68 | - |

Table S2. Linearly fitted rotational stiffness of the flexible hinges (N·mm/rad).

| Hinge number | #1 | #2 | #3 | #4 | #5 |
| --- | --- | --- | --- | --- | --- |
| Rotational stiffness | 6.722 | 6.829 | 6.807 | 6.749 | 6.722 |
| Hinge number | #6 | #7 | #8 | #9 |  |
| Rotational stiffness | 13.904 | 12.908 | 6.978 | 13.052 |  |

**Legends for Movies S1 to S4**

Movie S1.

The reconfiguration process of a 1×2 metamaterial among all physically possible configurations.

Movie S2.

The reconfiguration process of a 4×4 metamaterial among 10 configurations.

Movie S3.

Quasi-static uniaxial compression testing on a 4×4 TPU specimen.

Movie S4.

Real-time stiffness visualization of the 4×4 metamaterial.
